# Reprogramming mouse fibroblasts into engraftable myeloerythroid and lymphoid progenitors

**DOI:** 10.1038/ncomms13396

**Published:** 2016-11-21

**Authors:** Hui Cheng, Heather Yin-Kuan Ang, Chadi A. EL Farran, Pin Li, Hai Tong Fang, Tong Ming Liu, Say Li Kong, Michael Lingzi Chin, Wei Yin Ling, Edwin Kok Hao Lim, Hu Li, Tara Huber, Kyle M. Loh, Yuin-Han Loh, Bing Lim

**Affiliations:** 1 Stem Cell and Regenerative Biology Group, Genome Institute of Singapore, Singapore 138672, Singapore; 2 Epigenetics and Cell Fates Laboratory, Institute of Molecular and Cell Biology, Singapore 138673, Singapore; 3 Department of Biological Sciences, National University of Singapore, 14 Science Drive 4, Singapore 117543, Singapore; 4 Department of Molecular Pharmacology & Experimental Therapeutics, Center for Individualized Medicine, Mayo Clinic, Rochester, Minnesota 55905, USA; 5 Department of Developmental Biology, Stanford Institute for Stem Cell Biology and Regenerative Medicine, Stanford University School of Medicine, Stanford, California 94305, USA

## Abstract

Recent efforts have attempted to convert non-blood cells into hematopoietic stem cells (HSCs) with the goal of generating blood lineages *de novo*. Here we show that hematopoietic transcription factors *Scl*, *Lmo2*, *Runx1* and *Bmi1* can convert a developmentally distant lineage (fibroblasts) into ‘induced hematopoietic progenitors' (iHPs). Functionally, iHPs generate acetylcholinesterase^+^ megakaryocytes and phagocytic myeloid cells *in vitro* and can also engraft immunodeficient mice, generating myeloerythoid and B-lymphoid cells for up to 4 months *in vivo*. Molecularly, iHPs transcriptionally resemble native Kit^+^ hematopoietic progenitors. Mechanistically, reprogramming factor Lmo2 implements a hematopoietic programme in fibroblasts by rapidly binding to and upregulating the *Hhex* and *Gfi1* genes within days. Moreover the reprogramming transcription factors also require extracellular BMP and MEK signalling to cooperatively effectuate reprogramming. Thus, the transcription factors that orchestrate embryonic hematopoiesis can artificially reconstitute this programme in developmentally distant fibroblasts, converting them into engraftable blood progenitors.

## Abstract (web summary)

Direct reprogramming of closely-related lineages can generate hematopoietic stem cells. Here, the authors show hematopoietic transcription factors Scl, Lmo2, Runx1 and Bmi1 can reprogram fibroblasts into induced hematopoietic progenitors (iHPs), which are engraftable blood progenitors.

## Graphical abstract

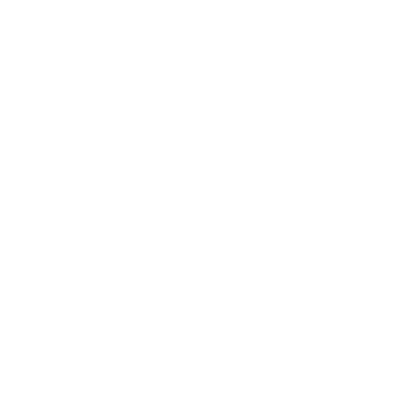

It is generally accepted that cellular identities are endowed by combinations of transcriptional regulators. Recent reports have shown that substitution of transcriptional regulators of one cell type with another's can rewrite cellular identity, thereby directly reprogramming one cell type into another^1^,^2^,^3^. Apart from the generation of induced pluripotent stem (iPS) cells from fibroblasts^4^, fibroblasts have also been directly reprogrammed into several ‘induced' lineages, such as cardiomyocytes, neurons and hepatocytes^5^,^6^,^7^,^8^.

Blood-forming hematopoietic stem cells (HSC) are amongst the most clinically-used adult stem cells. However, their use in the clinic is partially limited by the availability of matched bone marrow (BM) donors and the low frequency of stem cells in stored cord blood. Therefore alternative sources of HSCs are desirable. To this end, recent efforts have focused on directly converting various cell types into HSCs by overexpressing key hematopoietic transcription factors^9^.

Decades of research have revealed key transcription factors that are responsible for the specification, maturation and proliferation of HSCs during developmental ontogeny. In vertebrate embryos, transcription factors Scl, Lmo2 and Runx1 are required for the initial specification of HSCs from the mesoderm germ layer, presumably via a ‘hemogenic endothelium' intermediate^10^,^11^. Scl is one of the earliest-acting regulators of HSC specification and is critical for hemogenic endothelium specification, and Lmo2 normally acts as a bridge cofactor to Scl, whereas separately, core-binding factor Runx1 participates in a distinct transcriptional complex^11^,^12^,^13^,^14^,^15^,^16^. After their developmental specification, foetal and neonatal HSC self-renew due to the action of Sox17 (ref. 17). Subsequently in adulthood, Bmi1 (and potentially, Hoxb4) appear to regulate adult HSC self-renewal^18^,^19^. In sum, the implementation and perpetuation of the HSC programme is directed by an ordered series of transcription factors during both embryogenesis and adulthood.

Recently, it was reported that overexpression of a combination of transcription factors (HOXA9, ERG, RORA, MYB and SOX4) could drive human embryonic stem cell (ESC)-derived progeny into myeloerythroid progenitors that could engraft *in vivo*^20^. However, because these cells lacked robust lymphoid competence^20^, they seemingly corresponded to myeloerythroid precursors, not fully multipotent HSCs. More recently, differentiated mouse blood cells (for example, pro-B cells or granulocytes/monocytes) were ‘de-differentiated' into multipotent HSCs by a combination of eight transcription factors (Runx1t1, Hlf, Lmo2, Prdm5, Pbx1, Zfp37, Mycn and Meis1)^21^. Separately, FOSB, GFI1, RUNX1 and SPI1 were shown to reprogram human endothelial cells into engraftable hematopoietic multipotent progenitors (MPPs)^22^. While these were striking outcomes, differentiated blood progeny and endothelial cells already bear a close developmental affiliation with the HSC lineage. Thus, it was unclear whether these combinations of factors would suffice to enkindle a hematopoietic programme in completely unrelated cells such as fibroblasts.

Overexpression of Scl and Lmo2 sufficed to convert mouse fibroblasts into cells that could form hematopoietic colonies *in vitro*; however, the exact nature of these cells was not defined^23^. An expanded cocktail of transcription factors (Scl, Lmo2, Gata2, Runx1c and Erg) reportedly reprogrammed mouse fibroblasts into transiently-engrafting precursors that could generate myeloerythroid and lymphoid cells *in vitro* but exclusively generated TER119^+^ erythroid cells *in vivo* for a maximum of 2 weeks^24^. Finally, a partially-related cocktail of transcription factors (Gata2, Gfi1b, cFos and Etv6) successfully converted mouse fibroblasts into a hemogenic endothelium-like intermediate that could subsequently mature into colony-forming blood progenitors *in vitro*^25^; however, whether these cells could engraft *in vivo* remained unclear.

Collectively, HSCs can currently be generated from the direct reprogramming of closely-related lineages (either endothelial cells or more differentiated blood lineages)^21^,^22^, though the reconstitution of the HSC programme in developmentally distant lineages (for example, fibroblasts) has remained elusive. Given that current reprogramming regimens yield transiently-engrafting erythroid precursors from fibroblasts^24^, not multipotent hematopoietic stem/progenitor cells, this piques the question of what additional molecular machinery might endow the additional characteristic of prolonged *in vivo* self-renewal. Our efforts to this end have led to the identification of four hematopoietic transcriptional regulators (Scl, Lmo2, Runx1 and Bmi1 or alternatively, Scl, Lmo2, Runx1 and HoxB4) that can directly convert mouse fibroblasts into oligopotent hematopoietic progenitors. These reprogrammed hematopoietic progenitors have myeloid, erythroid and megakaryocyte differentiation potential *in vitro* and are capable of generating myeloid and B-lymphoid cells *in vivo* for up to 4 months in primary recipients. Furthermore, we examined mechanistic changes during such transdifferentiation to provide insight into how a completely non-hematopoietic programme may be reshaped into a hematopoietic phenotype. Reprogramming factor Lmo2 instills a hematopoietic programme within fibroblasts by binding to and upregulating the expression of critical hematopoietic factors (*Gfi1* and *Hhex*).

## Results

### Reprogramming fibroblasts to oligopotent blood progenitors

**Figure 1:**
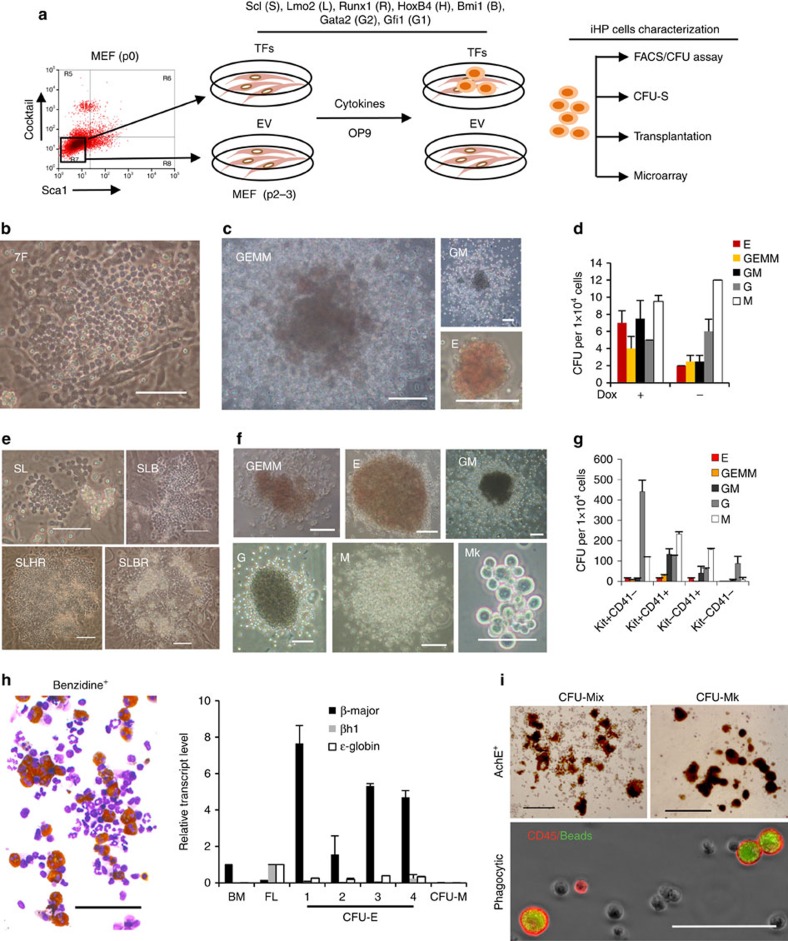
Generating induced hematopoietic progenitors from wild-type MEFs. (**a**) Schema of experimental design. MEFs (P0) were purified by sorting out any contaminating hematopoietic cells and passaged to P2-3 before experiments. iHP cells induced from MEFs by reprogramming factors were used for further characterization and evaluation; negative control cultures were transduced with empty vector (EV) only. (**b**) Representative ‘cobblestone' colonies at 24 dpi induced by lentiviral FuW-TetO vectors carrying 7F. Representative of three independent experiments. Scale bar: 100 μm. (**c**) CFU colonies derived from 27 dpi FuW-TetO-7F-induced iHP cells, representative of three independent experiments. Scale bar: 100 μm for GM, 50 μm for GEMM and E colonies. (**d**) Frequency of different type of CFU colonies derived from 27 dpi FuW-TetO-7F-induced iHP cells, with dox added (or withheld) at the beginning of CFC assays as indicated. Data are shown as mean±s.d. of four biological replicates from two independent experiments. (**e**) Representative ‘cobblestone' colonies (27 dpi) induced by different combinations of factors: SL, SLB, SLHR or SLBR (with factors singly delivered in individual pMX vectors), representative of three independent experiments. Scale bar: 100 μm. (**f**) Different types of representative CFU colonies derived from 27 dpi SLHR-iHP in CFC assays (factors were singly delivered in individual FuW-TetO vectors), representative of two independent experiments. Scale bar: 100 μm for GM/G colonies; 50 μm for GEMM/E/M/Mk colonies. (**g**) Frequencies of different types of colonies derived from FACS-sorted Kit^+^CD41^−^, Kit^+^CD41^+^, Kit^−^CD41^+^ and Kit^−^CD41^−^ subsets of SLHR-iHP cells in CFC assays. Factors were singly delivered in individual FuW-TetO vectors. Data shown are mean±s.d. of biological triplicates. (**h**) Benzidine positive cells in a GEMM colony and adult (*β-major*) globin expression in CFU-E colonies as shown in **f**, representative of two independent experiments. BM: bone marrow cells. FL: E12.5 foetal liver cells. Data shown are mean±s.d. of technical triplicates. Scale bar, 100 μm. (**i**) Images of AchE^+^ megakaryocyte-containing colonies (CFU-mix and CFU-Mk) and phagocytic CD45^+^ cells, representative of three independent experiments. 27 dpi SLHR-iHPs (induced using pMX vectors) were used for CFU-MK assay. Scale bar, 200 μm for CFU-mix/Mk colonies; 100 μm for phagocytosis picture.

In an effort to reprogram mouse embryonic fibroblasts (MEFs) into hematopoietic progenitors, we employed a reprogramming cocktail that included hematopoietic transcription factors with roles in either HSC specification or self-renewal. We started with a pool of seven well-characterized factors (henceforth, ‘7F'), namely *Scl* (S), *Lmo2* (L), *Runx1* (R), *HoxB4* (H), *Bmi1* (B), *Gfi1* (G1) and *Gata2* (G2)^26^,^27^ in *p53*^−/−^ MEFs, given indications that *p53*^−/−^ MEFs are more easily reprogrammed into iPSCs^28^ or blood progenitors^24^. Fibroblasts that were depleted of hematopoietic cells (Fig. 1a) were co-infected with the combination of seven hematopoietic factors and then seeded on inactivated OP9 feeder cells^29^ (Fig. 1a). 14–16 days post infection (dpi), small clusters of round cells could be distinguished from the flat fibroblasts (Supplementary Fig. 1a). By day 24, clear ‘cobblestone areas' were observed (Supplementary Fig. 1a), bearing resemblance to cobblestone area-forming units previously described for hematopoietic stem/progenitor cells^30^. Within another week, rounded cells were released from the cobblestone areas in large numbers into the medium. These cobblestone colonies contained cells expressing hematopoietic stem/progenitor markers Kit and CD41 (refs 31, 32; Supplementary Fig. 1a). Suspension cells showed characteristics of hematopoietic cells by FACS and morphological analyses (Supplementary Fig. 1b).

Fibroblasts, not residual endothelium^22^, were the starting cell type that was reprogrammed into hematopoietic cells. MEFs that were FACS depleted of CD31^+^ endothelial cells, or ear fibroblast cultures (which were totally devoid of CD31^+^ endothelial cells; Supplementary Fig. 2a), could both be reprogrammed into cobblestone-forming areas that released Kit^+^/CD41^+^ cells into suspension by 27 dpi (Supplementary Fig. 2a). In addition, there are no differences in the frequency of hematopoietic colonies (including clusters of round cells and cobblestone colonies) between CD31-depleted and CD31-undepleted MEFs (Supplementary Fig. 2a,b). These observations affirmed that hematopoietic cells could be induced from a developmentally distant lineage (fibroblasts) and that they did not emerge from residual endothelial cells.

Moreover wild-type fibroblasts (wild type for *p53*) could also be converted into hematopoietic cells; hence such reprogramming did not rely on *p53* deficiency. The 7F factors were overexpressed in wild-type MEFs, using constructs in which the transgenes were constitutively (pMX) or inducibly (FuW-TetO) expressed. Similarly to what was seen in a *p53*^−/−^ background, by 24 dpi clear ‘cobblestone areas' and rounded cells were induced by 7F in wild-type MEFs (Fig. 1b). These 7F-induced rounded cells were able to give rise to myeloid colonies in methylcellulose-based colony-forming cell (CFU-C) assays (Fig. 1c,d). Though slightly-red BFU/CFU-E (erythroid) colonies were observed when 7F were constitutively expressed (pMX vector; not shown), if transgene expression was discontinued at the start of the CFU assay (by withdrawing doxycycline at the beginning of the CFU assay; FuW-TetO vector), visibly red CFU-GEMM or BFU/CFU-E emerged (Fig. 1c,d). This suggests that continued transgene expression can impair erythroid differentiation *in vitro*. Finally, though induced hematopoietic cell reprogramming was fully successful in wild-type cells, suppression of *p53* led to a ∼10-fold enhancement in efficiency as shown by overexpression of a dominant-negative *p53* construct (p53DD)^33^ (Supplementary Fig. 2c).

In sum, wild-type MEFs could be reprogrammed into hematopoietic cells with progenitor characteristics that could form both ‘cobblestone' areas and CFU colonies. We refer to these cells as induced hematopoietic progenitors (iHP), and further interrogated the prerequisites for their generation.

### *HoxB4* or *Bmi1* enhances iHP induction atop *Scl* and *Lmo2*

To determine which of the seven factors were crucial for generating iHPs, we evaluated the effect of omitting one gene at a time in a *p53*^−/−^ background. Strikingly, the exclusion of either Scl (S) or Lmo2 (L) completely abrogated the generation of any iHP cells. Individually removing other factors did not block the appearance of cobblestones or Kit^+^ cells (Supplementary Fig. 1c). Furthermore, all 7F iHP-derived CFU colonies examined contained integration of the S and L transgenes (Supplementary Fig. 2d), further indicating that both S and L are absolutely necessary for iHP reprogramming.

Accordingly, in wild-type MEFs, S and L together sufficed to generate ‘cobblestone areas' that released Kit^+^ hematopoietic progenitors into the suspension, corroborating that S and L are sufficient to reprogram fibroblasts into hematopoietic CFUs^23^,^24^ (Fig. 1e, Supplementary Fig. 2e). Atop the rudimentary background of S and L, the further inclusion of *HoxB4* (H) or *Bmi1* (B) or together with *Runx1* (R) led to a ∼10-fold increase in the frequency of Kit^+^ cells in suspension (Fig. 1e, Supplementary Fig. 2e).

### Kit^+^ cells are enriched for iHP progenitor activity

Expressing this optimized quartet of *Scl*, *Lmo2*, *Runx1* and *Hoxb4* using a doxycycline-inducible construct also generated iHPs (SLHR-iHPs wherein factors S, L, H, R were singly delivered in individual vectors) that harboured the ability to form CFU-GEMM as evinced by *in vitro* CFU-C assays (Fig. 1f–g) and CFU-mix colonies within *in vitro* collagen-based CFU-Mk assays in the presence of dox (Supplementary Fig. 2g).

**Figure 2:**
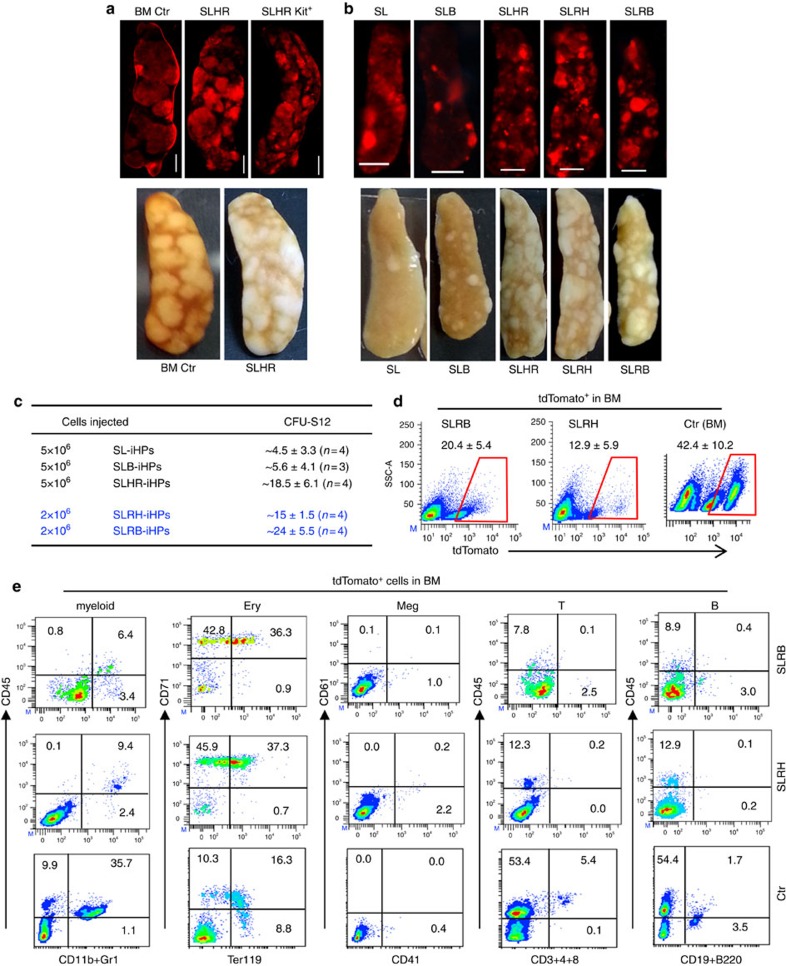
*Runx1* together with *HoxB4* or *Bmi1* augments the CFU-S forming-ability of iHP cells. (**a**) Similar to control bone marrow cells (BM Ctr, tdTomato^+^), SLHR-iHP cells (tdTomato^+^) form tdTomato^+^ nodules in the spleen at 12 days post-transplantation (dpt) into lethally irradiated C57Bl/6 mice (CFU-S12). Factors were singly delivered in individual pMX constructs. 1 × 10^5^ BM control cells, 5 × 10^6^ SLHR-iHP cells or 1 × 10^6^ Kit^+^ cells were transplanted per mouse. Mice analysed: BM Ctr, *n*=4, SLHR-iHP cells, *n*=20, SLHR-iHP kit^+^ cells, *n*=4. These are representative of three independent experiments. Scale bar, 2 mm. (**b**) Comparison of CFU-S12 potential of iHP cells induced by differing TF cocktails. SL-, SLB-, SLHR-, SLRH- or SLRB-iHP cells (tdTomato^+^) were transplanted into lethally irradiated SCID mice. Factors were delivered in pMXconstructs; SLHR denotes individual delivery of S, L, H and R; SLRH and SLRB denotes use of a polycistronic construct containing S, L and R in one pMX vector, together with individual delivery of either H or B in a separate construct. For SL-, SLB-, SLHR-iHP, 5 × 10^6^ cells were transplanted. For SLRH- and SLRB-iHPs, 2 × 10^6^ cells were transplanted. Mice analysed: for SL-, SLB-, SLHR-iHP, *n*=6, for SLRB- and SLRH-iHP, *n*=12 each. Scale bar: 2 mm. These are representative of three independent experiments. (**c**) Frequency of CFU-S12 of different iHP cells. iHP cells are named as in **b**. Data are shown as mean±s.d. per spleen. These data are from three independent experiments. (**d**) Representative FACS analysis of tdTomato^+^ cells in BM of SCID mice at 12–14 dpt. SLR factors were delivered in one polycistronic construct. For SLRB/H-iHP cells: 2 × 10^6^ cells per mouse were transplanted; for Ctr BM cells (tdTomato^+^), 2 × 10^5^ cells per mouse were transplanted. Percentage of tdTomato^+^ cells are shown as mean±s.d. (*n*=6 mice for each type of cells), representative from three independent experiments. (**e**) Representative FACS analysis of tdTomato^+^ cells stained with lineage markers (shown on the plots) in the BM of SCID mice transplanted with either SLRB/SLRH-HP cells and control BM cells (Ctr tdTomato^+^) at 12–14dpt. SLR factors were delivered in one polycistronic construct. Mice analysed: *n*=6 for each type of cells. Ery, Erythroid; Meg, Megakaryocytes. These data are representative of three independent experiments.

Given that surface markers Kit and CD41 enrich for colony-forming progenitors in embryonic hematopoiesis^34^, we assessed the *in vitro* colony-forming activity of four cell populations (Kit^+^CD41^−^, Kit^+^CD41^+^, Kit^−^CD41^+^, Kit^−^CD41^−^) sorted from SLHR-iHP cultures (Supplementary Fig. 2f). Kit^−^CD41^−^ cells only contained limited myeloid progenitor potential, while multipotent and committed progenitors were enriched in the Kit^+^ fraction (either CD41^−^ or CD41^+^; Fig. 2g). Similarly in the collagen-based CFU-Mk assay, Kit^+^CD41^+^ progenitors could form CFU-mix containing both myeloid lineages (G/M) and megakaryocytes (Mk or Meg). By contrast, the Kit^−^CD41^+^ fraction harboured more committed megakaryocyte progenitor activity (Supplementary Fig. 2g). Collectively this demonstrates that oligopotent myeloerythroid progenitor activity is contained within Kit^+^ iHPs.

The myeloerythroid progeny of SLHR-iHPs displayed characteristics of mature, differentiated cells. In CFU assays, visibly red colonies were derived from SLHR-iHPs (evincing their production of haemoglobin; Fig.1f, h) and moreover these colonies expressed definitive ‘adult-type' haemoglobin genes (for example, β-globin), over foetal (βH1) or embryonic (ɛ-globin) genes (Fig. 1h). This was striking, considering that ESC-derived erythroid cells typically display a more immature (foetal/embryonic) globin pattern. Furthermore, SLHR-iHPs produced megakaryocyte-containing colonies that were strongly reactive for acetylcholinesterase (Fig. 1i), which is a marker of more mature megakaryocytes. In addition, SLHR-iHP-derived CD11b^+^ and Gr-1^+^ cells (corresponding to granulocytes and/or monocytes) could phagocytose fluorescently-labelled latex beads, but iHP-derived Kit^+^ cells failed to do so (Fig. 1i, Supplementary Fig. 2h). Therefore, SLHR-iHP myeloerythroid derivatives displayed mature functionalities consistent with their surface marker identities, providing evidence that iHPs harbour myeloerythroid potential.

### *Runx1* is critical to generate iHPs that form CFU-S

iHP also engrafted *in vivo*, and upon transplantation into irradiated mice generated Colony-Forming Units in the Spleen (CFU-S)—a short-term quantitative *in vivo* assay for hematopoietic stem and progenitor cells^35^. To this end, we used constitutively-active pMX vectors to reprogram constitutively tdTomato-expressing MEFs (wild type for *p53*) and transplanted the resultant iHPs into irradiated SCID and C57BL/6 recipients, allowing us to trace their *in vivo* contribution to tdTomato^+^ progeny. SL-iHPs formed a diffuse distribution of tdTomato^+^ CFU-S nodules in the spleen (SP). However, the inclusion of Runx1 (R) and Hoxb4 (H) during reprogramming instilled SLHR-iHPs with a quantitatively 4-fold greater ability to form CFU-S (Fig. 2a–c). In addition, SLHR-iHPs formed CFU-S nodules that were larger than the ones derived from SL, SLB or SLH-iHPs (Fig. 2b, Supplementary Fig. 3a,b), indicating the importance of R and H in enhancing *in vivo* engraftment. CFU-S activity was enriched five-fold in the Kit^+^ fraction of SLHR-iHP cells (Fig. 2a), consistent with the notion that iHP progenitor activity is enriched in the Kit^+^ population.

The use of an ‘all-in-one' polycistronic construct carrying three of the four factors (‘SLR': S, L and R linked together by 2A sequences) further increased the CFU-S efficiency of SLRH-iHPs by 2-fold as compared with SLHR-iHPs wherein each of the four factors was individually delivered (Fig. 2c). SLRB-iHPs (where polycistronic SLR was used, *Bmi1* in a separate construct was used in lieu of *HoxB4*) also harboured robust CFU-S activity *in vivo* (Fig. 2b,c). Altogether, though S and L are minimally sufficient to generate iHPs, the ability of such progenitors to form CFU-S *in vivo* is augmented by the inclusion of R and either B or H.

In iHP-transplanted mice that were killed at early timepoints (12–14 days post-transplant [dpt]), iHP-derived tdTomato^+^ cells also contributed to the BM and the peripheral blood (PB; Fig. 2d, Supplementary Fig. 3c,d). The majority of tdTomato^+^ cells were TER119^+^ erythroid cells or CD45^+^/Gr-1^+^/CD11b^+^ which were myeloid cells (CD11b^+^Gr1^low/−^ or CD11b^+^Gr1^high^). A very small population of CD61^+/low^/CD41^+^ putative megakaryocytes was also detected. Finally, B220^+^CD19^+^ B lineage cells were low but detectable in SLRB-iHP injected mice at this early stage (Fig. 2e, Supplementary Fig. 3e).

### SLRB-iHPs engraft for up to 4 months *in vivo*

The *sine qua non* of hematopoietic stem/progenitor cells is their ability to reconstitute myeloerythroid and lymphoid lineages in irradiated mice. Since *in vitro* (Fig. 1) and *in vivo* (CFU-S; Fig. 2) assays indicated that SLRB- and SLRH-iHPs harboured hematopoietic progenitor activity, we sought to determine whether iHP cells (wild type for *p53*) were indeed capable of long-term repopulation of the hematopoietic system in an irradiated mouse.

**Figure 3:**
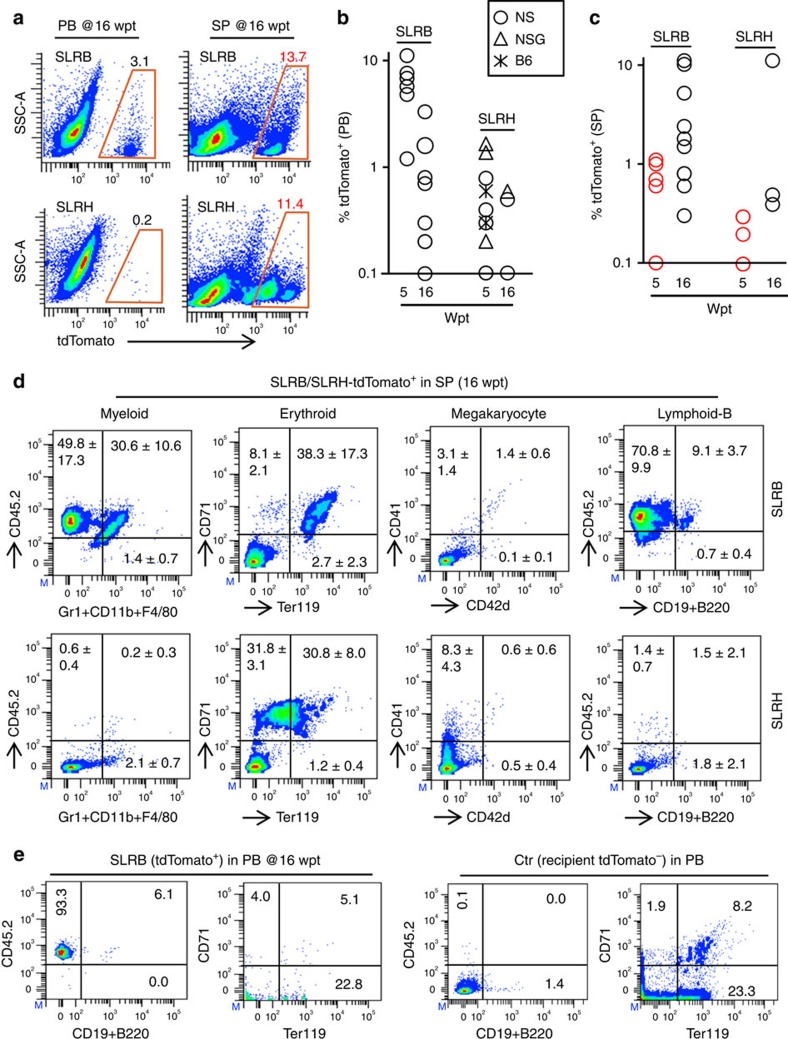
SLRB-iHP cells engraft for up to 4 months *in vivo*. (**a**) Representative FACS plot of SLRB/SLRH-iHP (tdTomato^+^) cells in PB and SP at 16 wpt (Wpt: week post-transplant). SLR factors were delivered in one polycistronic construct. 5 × 10^6^ iHP cells were transplanted per mouse. Mice analysed: SLRB iHP (*n*=9), SLRH-HP (*n*=4). These are representative of three independent experiments. (**b**) Summary of SLRB/SLRH-iHP (tdTomato^+^) cell engraftment in PB at 5 and 16 wpt. SLR factors were delivered in one polycistronic construct. For SLRB-iHP, NOD-SCID (NS) mice *n*=6 (5 wpt) and *n*=9 (16 wpt) were analysed. For SLRH-iHP, different mouse strains were analysed. At 5wpt, NS mice *n*=5, NSG mice *n*=3, C57BL/6 (B6) mice *n*=3. At 16 wpt, NS mice *n*=3, NSG mice *n*=1, B6 mice *n*=2. Only tdTomato^+^ cell contribution ≥0.1% are plotted. Data drawn from three independent experiments. (**c**) Summary of SLRB/SLRH-iHP (tdTomato^+^) cell engraftment in SP at 5 and 16 wpt. SLR factors were delivered in one polycistronic construct. For SLRB-iHP cells, mice *n*=5 at 5wpt, and *n*=9 at 16 wpt. For SLRH-iHP cells, mice analysed: *n*=5 at 5wpt, *n*=4 at 16 wpt. Only tdTomato^+^ cells contribution ≥0.1% were plotted. These data are from three independent experiments. (**d**) Representative FACS plot of multilineage reconstitution of SLRB/H-iHP (tdTomato^+^) cells in SP at 16 wpt. SLR factors were delivered in one polycistronic construct. Percentage of cells are shown as mean±s.d. (for SLRB, *n*=6 mice; for SLRH, *n*=3 mice). These data are representative of three independent experiments. (**e**) SLRB-iHP (tdTomato^+^) cells in PB at 16 wpt. SLR factors were delivered in one polycistronic construct. tdTomato^−^ PB cells from respective CD45.1^+^ recipient mice shown as control (Ctr). These data are representative of three independent experiments.

Upon transplantation into irradiated NOD-SCID (NS) mice, both SLRH-iHP and SLRB-iHP (in which transgenes were constitutively expressed) contributed to all hematopoietic organs—PB (Fig. 3a,b, Supplementary Fig. 4a), SP (Fig. 3a, c, Supplementary Fig. 4a) and BM (Supplementary Figs. 4a and 5a–c)—for up to 16 weeks. SLRB-iHPs were significantly more robust at PB reconstitution at 5 weeks post-transplant (wpt) and 16 wpt by comparison with SLRH-iHP (Fig. 3a,b, Supplementary Fig. 4a). In contrast, SLRH-iHP showed higher BM engraftment at 16 wpt than SLRB-iHP (Supplementary Fig. 5a–c).

Examination of donor cell contribution in SP, PB and BM at 5wpt and 16 wpt revealed SLRB-iHPs differentiated into CD45^+^/CD11b^+^/Gr1^+^/F4/80^+^ myeloid cells, CD45^+^/B220^+^/CD19^+^ B-lymphoid cells as well as Ter119^+^ erythroid cells *in vivo*. CD41^+^ or CD61^+/low^ megakaryocytes were predominately detected in the SP instead of BM at 5 wpt (Fig. 3d,e, Supplementary Fig. 4b–d), as previously noted after HSC transplantation into irradiated mice^36^. SLRH-iHPs contributed to multiple lineages at 5 wpt, but mainly contributed only to Ter119^+^ erythroid cells and CD41^+^/CD42d^+^ megakaryocytes at 16 wpt (Fig. 3d, Supplementary Figs 4b–d and 5d). Hence, the inclusion of *Bmi1* (B) is instrumental to endow iHPs with more robust *in vivo* reconstitution potential and the capacity to differentiate into B-lymphoid cells, potentially paralleling the known role of *Bmi1* in HSC/progenitor self-renewal^18^,^37^,^38^.

**Figure 4:**
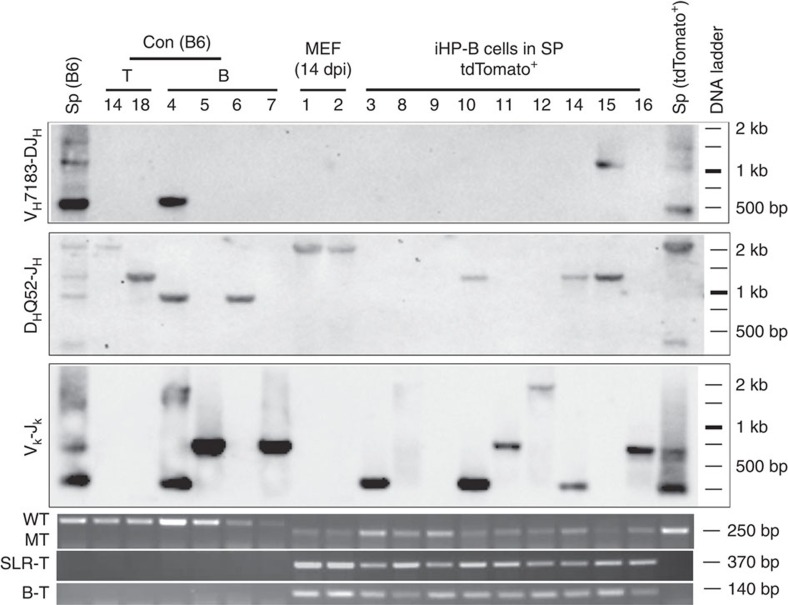
SLRB-iHPs generate B cells *in vivo*. V(D)J recombination events and transgene integration were detected in SLRB-iHP-derived B cells (iHP-B cells) at single-cell level. iHP-B cells are from 16 wpt spleens. Numbers at top of the panel are cell ID of individual single cells. Con (B6) denotes control (Con) cells from the SP of C57BL/6 (B6) mice. MEF (14 dpi) are cells from SLRB infected tdTomato^+^ MEF at 14 dpi. Sp (B6) and Sp (tdTomato^+^) stand for total spleen cells. V_H_7183-DJ_H_ and D_H_Q52-J_H_ are used to reveal V to DJ and D to J recombination events at heavy chain, respectively. V_k_−J_k_ denotes V to J recombination at Kappa chain. WT (Wild type) and MT (tdTomato mutant) show the genotype of the cells. SLR-T and B-T stand for transgene integration of SLR (Polycistronic) and Bmi1. Polycistronic SLR was used for the generation of iHP-B cells and 14 dpi MEF. These are representative of two independent experiments.

To further test the authenticity of iHP-derived B-lymphoid cells (wild type for *p53*), we assessed at their ability to undergo DNA recombination at the *IgH* and *IgL* gene loci (known as V(D)J recombination) at the 16 wpt timepoint. Recombination events were detected in iHP-derived B cells at either the heavy chain or kappa chain genes at single-cell level (Fig. 4, Supplementary Fig. 10). When genotyped, all the donor single cells were homozygous for the tdTomato knock-in and integrations of both *SLR* (polycistronic) and *Bmi1* transgenes were detected in the genome, confirming that they were indeed derived from iHP cells (Fig. 4, Supplementary Fig. 10).

Furthermore, CFC assay of total BM cells from 16 wpt mice originally engrafted with SLRB-iHPs or SLRH-iHPs indicated that even at this late timepoint, there were donor cells that still retained hematopoietic progenitor activity (Supplementary Fig. 5e,f). However, iHPs were not significantly detected in the PB at 5wpt after secondary transplantation (not shown). Indeed, BM-derived self-renewing lineage-restricted progenitors and MPPs contribute to multilineage reconstitution in primary recipients but not after serial transplantation^39^, and hence iHPs might broadly approximate either of these lineages. Though the exact endogenous BM counterpart to iHP remains unclear, we exploited this hematopoietic reprogramming system to uncover molecular mechanisms by which a hematopoietic phenotype may be imposed on a developmentally-distal lineage; namely, fibroblasts.

### Transcriptional and genomic analysis of iHP reprogramming

**Figure 5:**
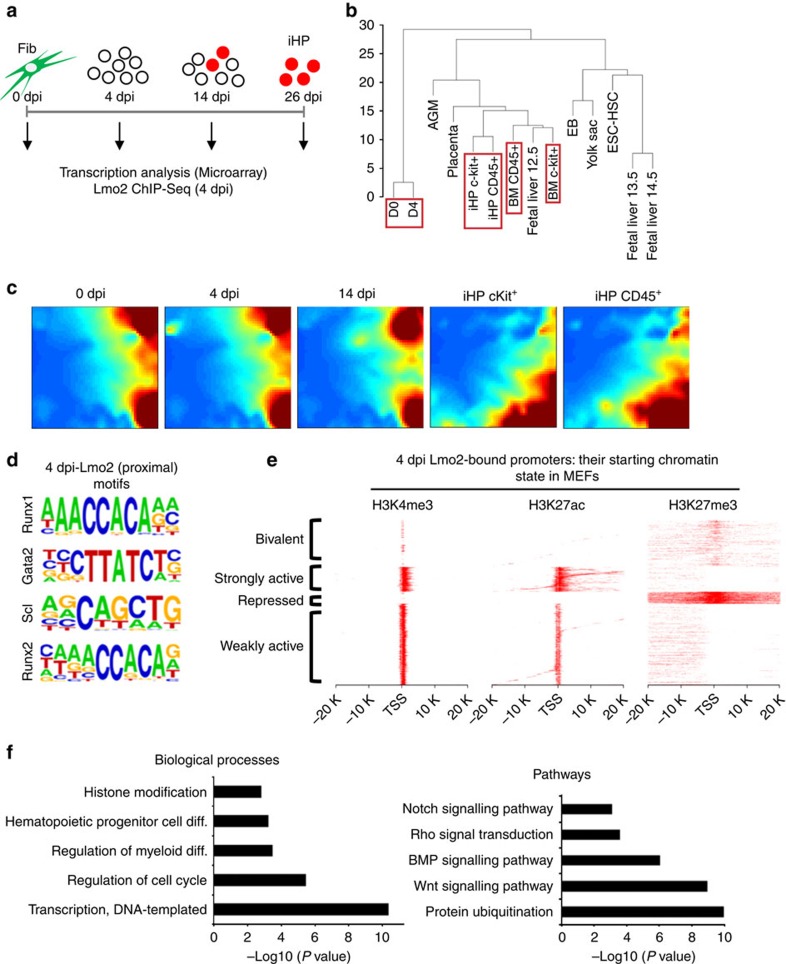
Transcriptional dynamics and Lmo2 genomic binding during iHP reprogramming. (**a**) Schema of the transdifferentiation procedure with days indicating when samples were collected for microarray and ChIP-Seq analyses. (**b**) Hierarchical clustering of D0, D4, iHP (Kit^+^ and CD45^+^ cells) and BM (Kit^+^ and CD45^+^ cells) with published HSPC microarray datasets^40^. Red boxes denote the samples generated in this study whereas the other samples are HSPCs from published datasets^40^. In brief, HSPCs from published datasets are VE-cadherin^+^CD45^+^ cells in AGM, Lin^−^Sca1^+^Kit^+^VE-cadherin^+^Mac-1^low^ cells in E12.5 foetal liver, CD45^+^Kit^+^CD34^mid^ cells in E12.5 placenta, Lin^−^Sca1^+^Kit^+^CD150^+^CD48^−^ cells in E13.5 and E14.5 foetal liver, CD41^+^Kit^+^CD34^+^ cells in yolk sac, Kit^+^CD41^+^ cells in day 6 embryoid bodies (EB), and CD41^bright^CD45^−^CD34^−^ ESC-derived HSC-like cells. (**c**) Dynamic gene expression during the microarray timecourse, as assayed by GEDI. The GEDI plots demonstrate a transition state in 14 dpi cells and a major change of the gene expression profile at 26 dpi cells during reprogramming. (**d**) Motif analysis for Lmo2-bound peaks at 4 dpi shows an enrichment of motifs recognized by other hematopoietic transcription factors. (**e**) Clustered heatmaps of MEF H3K4me3, H3K27ac and H3K27me3 signals at genomic regions bound by Lmo2 at 4 dpi. The heatmaps indicate that 4 dpi Lmo2 binds both closed and open chromatin. (**f**) GO analysis of Lmo2-bound genes; bar plots indicate *P* values of the overrepresented pathways and biological processes.

To evaluate the ordered series of molecular events driving fibroblasts to a hematopoietic fate, we performed microarray transcriptome analysis of tdTomato^+^ fibroblasts (MEFs), intermediate populations undergoing SLHR reprogramming (4 dpi and 14 dpi), reprogrammed 26 dpi iHPs (CD45^+^ or Kit^+^) and finally BM (CD45^+^ or Kit^+^) cells as a positive control (Fig. 5a). We also profiled the genome-wide binding of transcription factor Lmo2 at an incipient stage of SLHR reprogramming (namely, 4 dpi; Fig. 5a).

Expression profiling revealed that iHPs (CD45^+^ or Kit^+^) clustered closely with adult BM CD45^+^ and Kit^+^ cells, while D0 fibroblasts (0 dpi) and D4 (4 dpi) cells clustered away from other cells (Fig. 5b, Supplementary Fig. 6a,b). When HPSCs of various embryonic stages (taken from a published dataset^40^) were included in the clustering analysis, our iHPs also cluster closely with Kit^+^CD34^mid^CD45^+^ placental HSPCs and Lin^−^Sca1^+^Kit^+^ foetal liver HSPCs (Fig. 5b), affirming the hematopoietic progenitor identity of our iHP cells. To examine the transcriptional timecourse through which such hematopoietic reprogramming occurred, we employed Gene Expression Dynamic Inspection (GEDI)^41^ and indeed found that pronounced transcriptional changes began to occur at 14 dpi (Fig. 5c),

Mechanistically, the hematopoietic reprogramming factors should implement a hematopoietic identity in fibroblasts by binding to and either activating (or repressing) a suite of target genes within chromatin. Therefore we assayed the genome-wide binding of transcription factor Lmo2, given the primacy of Lmo2 in driving iHP reprogramming, and its known role as a bridging molecule assembling a multi-member hematopoietic transcription factor complex^16^,^42^. Indeed analysis of Lmo2-bound chromatin at 4 dpi revealed that such DNA was also enriched for motifs recognized by other hematopoietic transcription factors (for example, Runx, Scl and Gata factors; Fig. 5d, Supplementary Fig. 6c,d), similar to the situation seen for Lmo2 in hematopoietic progenitor cells^42^.

Could Lmo2 directly access target genes in closed chromatin within fibroblasts *à la* a pioneer factor^43^, or could it only bind pre-existing open chromatin? It should be noted that transcription factor Lmo2 does not have a DNA-binding domain but serves to nucleate complexes between a variety of transcription factors and chromatin remodelers^16^; therefore while Lmo2 itself might not be a pioneer factor *sensu stricto*, Lmo2-containing transcriptional regulatory complexes might harbour pioneer factor activity. To answer this question, for Lmo2-bound promoters (peaks surrounding the TSS) at 4 dpi, we analysed the *ab initio* chromatin states of these loci within MEFs before reprogramming using published data^44^. Analyses of H3K4me3, H3K27ac and H3K27me3 enrichment indicated that Lmo2 could bind to closed chromatin: Lmo2 could access bivalent (H3K4me3^+^/H3K27me3^+^) and repressed (H3K27me3^+^) promoters in MEFs (Fig. 5e, Supplementary Fig. 6e). As expected, Lmo2 could also bind to promoters that were also already active (H3K4me3^+^, with different extents of H3K27ac enrichment; Fig. 5e, Supplementary Fig. 6e). Gene Ontology analysis of Lmo2-bound genes indicated an enrichment of genes associated with cell cycle, transcriptional regulation, histone modifications and hematopoietic cell fate determination. In addition, pathway analysis revealed Lmo2-bound genes involved in protein ubiquitination and signalling pathways such as the bone morphogenetic protein (BMP) family, Wnt, Notch and Rho cascades (Fig. 5f, Supplementary data 1).

### Lmo2 implements iHP reprogramming through *Hhex* and *Gfi1*

**Figure 6:**
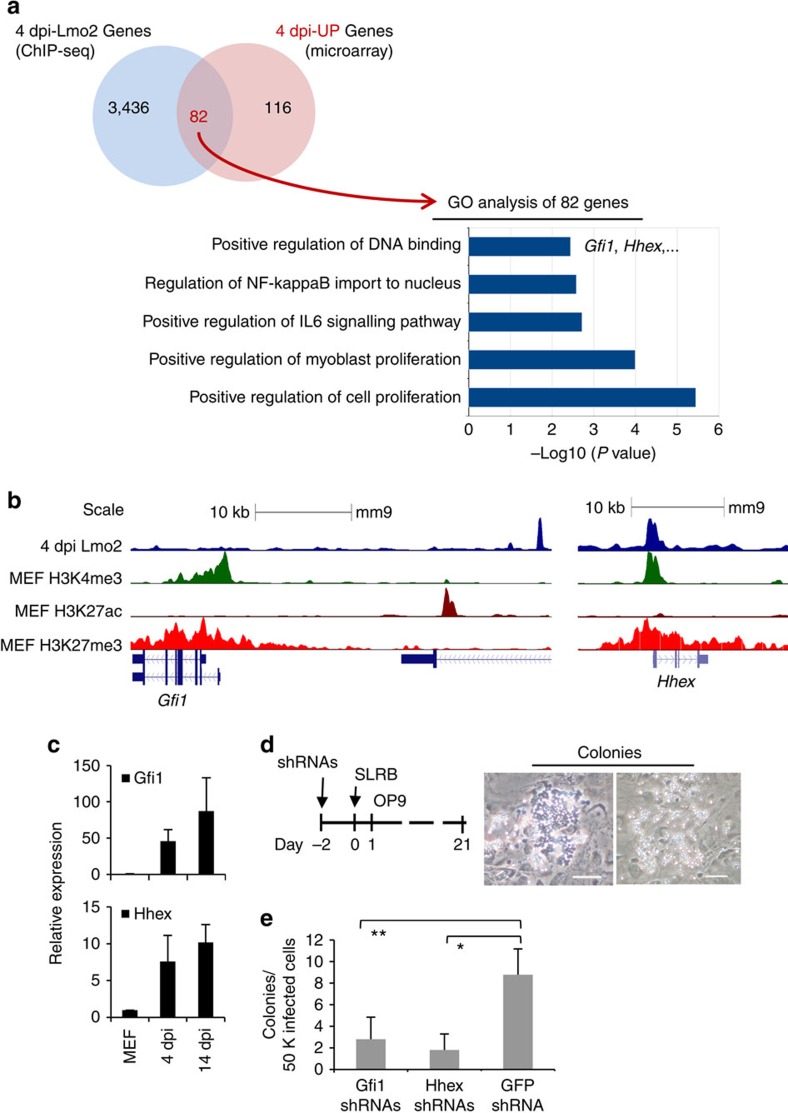
Lmo2 binds to *Hhex* and *Gfi1* to effectuate iHP reprogramming. (**a**) Venn diagram demonstrating the numbers of 4 dpi Lmo2-bound genes that are upregulated at 4 dpi cells and GO analysis of the 82 genes that both Lmo2-bound and 4 dpi upregulated. (**b**) Mouse genome screenshots demonstrating binding of Lmo2 on the *Gfi1* enhancer^70^ and the *Hhex* promoter at 4 dpi. (**c**) *Gfi1* and *Hhex* were upregulated at early stages of SLRB-iHP reprogramming; SLR factors were delivered in one polycistronic construct. Data shown are mean±s.d. of technical triplicates, representative of two independent experiments. (**d**) shRNAs against *Hhex* or *Gfi1* were introduced into fibroblasts 2 days before introducing the SLRB reprogramming factors; SLR factors were delivered in one polycistronic construct. Images are hematopoietic colonies representative of five independent experiments. Scale bar: 100 μm. (**e**) Knockdown of *Gfi1* or *Hhex* perturbs iHP reprogramming. 50,000 (50 K) MEFs were infected with SLRB and hematopoietic colonies (including cobblestone colonies and clusters of round cells, as shown in **d**) were enumerated at 21 dpi. SLR factors were delivered in one polycistronic construct. Data shown are mean±s.d. of five independent experiments. *GFP* shRNA was used as a non-targeting control; *P<0.001, **P<0.003 (two-tailed Student's *t*-test).

When the hematopoietic reprogramming factors bind to the fibroblast genome, they must upregulate a cohort of critical genes beyond themselves to implement blood-cell fate (Supplementary Fig. 7). Intersecting 4 dpi Lmo2-bound genes with microarray data of genes upregulated or downregulated in 4 dpi revealed a number of potential Lmo2-targeted genes including those involved with ‘positive regulation of cell cycle' and ‘positive regulation of DNA binding' such as *Hhex* and *Gfi1* (Fig. 6a,b, Supplementary Fig. 8a, c, Supplementary Table 1, Supplementary data 2&3). Both *Hhex* and *Gfi1* are amongst the earliest markers of definitive hematopoiesis and critical for hematopoietic development as evinced by their knockout phenotypes in the mouse embryo^45^,^46^,^47^. Since *Hhex* and *Gfi1* were Lmo2-bound and transcriptionally upregulated at 4 dpi (Fig. 6a–c, Supplementary Fig. 9a), we tested the effects of knocking down *Hhex* and *Gfi1* in fibroblasts using shRNAs 2 days before introducing the SLRB reprogramming factors. Knockdown of either *Hhex* or *Gfi1* led to a strong reduction in the number of hematopoietic colonies that formed after 20 days of reprogramming (Fig. 6d,e, Supplementary Fig. 9b). This therefore suggests that Lmo2 recruits transcriptional machinery involved in embryonic hematopoiesis (for example, Hhex and Gfi1) to collectively effect hematopoietic reprogramming.

### BMP and MAPK/ERK cascades are required for iHP formation

**Figure 7:**
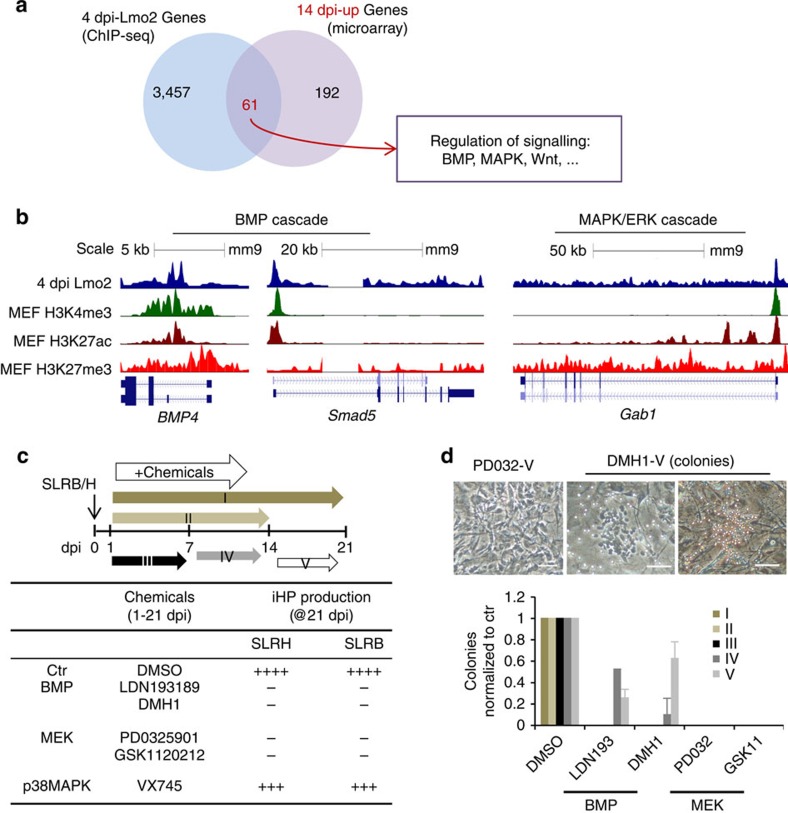
BMP and MAPK/ERK signalling cooperate to drive iHP reprogramming. (**a**) Venn diagram demonstrating the numbers of 4 dpi Lmo2-bound genes that were upregulated in 14 dpi cells. The box demonstrates enrichment of genes associated with various signalling pathways that both Lmo2-bound (4 dpi) transcriptionally upregulated (14 dpi). (**b**) Mouse genome screenshots demonstrating the binding of 4 dpi Lmo2 onto the promoters of various signalling components (for example, *Bmp4, Smad5,* BMP4 cascade; *Gab1*, MAPK-MEK cascade). These are representatives from a single experiment. (**c**) Addition of signalling pathway modulators during SLRB iHP reprogramming reveals that specific inhibition of either BMP or MEK pathways abrogates iHP formation; SLR factors were delivered in one polycistronic construct. LDN193189 (0.4 μM), DMH1 (1 μM), PD0325901 (0.8 μM) and GSK1120212 (0.4 μM) were added every other day from 1–21 dpi. These data are from four independent experiments. (**d**) BMP signalling is critical for iHP reprogramming at early stage (from 1–7 dpi), while MEK signalling is required throughout SLRB iHP reprogramming. SLR factors were delivered in one polycistronic construct. Pictures shown exemplify hematopoietic colonies observed at 21 dpi. The effects of various small-molecule inhibitors on hematopoietic colony formation was normalized to DMSO-treated control cultures (21 dpi). Images are representative of four independent experiments. Scale bar: 100 μm. Data shown were mean±s.d. of four independent experiments. Chemicals were added at different time windows (I, II, III, IV and V) as diagrammed in **c**.

By overlapping genes that are bound by Lmo2 at 4 dpi and that are transcriptionally upregulated or downregulated at 14 dpi, we found genes that were associated with several extracellular signalling pathways such as the BMP and mitogen-activated protein kinase (MAPK) cascades (Fig. 7a, Supplementary Fig. 8b,d,e, Supplementary Table 1, Supplementary data 4&5)^48^, which have a known role in HSC specification during vertebrate ontogeny. Known BMP signalling regulators *Bmp4* and *Smad5* and MAPK cascade regulator *Gab1* were all bound by Lmo2 at 4 dpi (Fig. 7b). A pathway map of the MAPK pathway demonstrated the extent to which elements of this pathway are pervasively bound by Lmo2 (Supplementary Fig. 9c), in particular the MAPK/ERK (extracellular signal-regulated kinases) (MEK) cascade.

To test whether the BMP and MAPK/ERK pathways were functionally required for iHP reprogramming, highly-specific pharmacologic inhibitors of these pathways were added to reprogramming cultures at different timepoints (schema in Fig. 7c). Strikingly, small-molecule inhibition of BMP signalling from 1–21 dpi using two independent inhibitors, either LDN-193189 or DMH1, completely inhibited the formation of hematopoietic colonies (Fig. 7c). Similarly, blockade of MEK signalling from 1 to 21 dpi using structurally-unrelated inhibitors (PD0325901 or GSK1120212) abolished iHP generation (Fig. 7c). By contrast small-molecule inhibitors of p38 MAPK did not significantly impair reprogramming (Fig. 7c).

Having established that long-term blockade of either BMP or MEK signalling completely abrogated iHP formation, we determined the specific timepoints at which these developmental signals were needed. BMP activity was most critical during the first 7 days of iHP reprogramming, as later-stage inhibition still permitted the formation of iHPs, albeit at a reduced frequency (Fig. 7d). By contrast MEK inhibition at any timepoint fully extinguished iHP generation, indicating that its continuous signalling is required to drive iHP induction (Fig. 7d). These data indicate that aside from the transcriptional control asserted by the reprogramming transcription factors, extracellular BMP and MEK signalling is also required to cooperatively execute reprogramming.

## Discussion

Here we show that the combined action of four transcription factors (*Scl*, *Lmo2*, *Runx1* and *Bmi1*) can decisively redirect the fate of mouse fibroblasts, reprogramming them into engraftable hematopoietic progenitors (iHPs). The resultant hematopoietic progenitors were capable of reconstituting myeloerythroid and B-lymphoid lineages *in vivo* for up to 4 months in primary recipients, but not in secondary recipients.

Earlier work established that committed hematopoietic progeny (for example, pro-B cells or myeloid cells) could be ‘de-differentiated' into HSCs^21^ and that a hematopoietic-related lineage (endothelial cells) could be reprogrammed into HSCs^22^. However, since hematopoietic progeny and endothelium are cell types that are already closely affiliated with the HSC lineage, whether a completely developmentally unrelated lineage could be respecified to a hematopoietic fate remained unclear. Though fibroblasts could be reprogrammed into short-term erythroid precursors using five transcription factors (*Scl*, *Lmo2*, *Runx1c*, *Gata2* and *Erg*)^24^, this finding left unanswered two key questions: how might a multipotent state be synthesized and how more long-term self-renewal might be molecularly endowed.

Starting with seven well-known hematopoietic factors, we showed that a combination of Scl and Lmo2 was sufficient to recommit mouse fibroblasts into hematopoietic progenitors. *Scl* and *Lmo2* are coexpressed in the earliest hematopoietic mesoderm in the vertebrate embryo^14^. Pertinently, overexpression of *Scl* and *Lmo2* can convert non-axial mesoderm into hematopoietic mesoderm in zebrafish embryos^49^ and can also confer hematopoietic characteristics upon mouse fibroblasts^23^,^24^. It is noteworthy that enforced co-expression of *Scl* and *Lmo2* was able to induce a lineage transition from fibroblast to blood in a dish—therefore these factors constitute early-acting ‘lineage-instructive factors' both in embryogenesis and during lineage reprogramming. Furthermore, Lmo2 itself has no DNA-binding domain but crucially acts as a ‘bridge factor' that physically bridges Scl to a large cohort of transcriptional regulators in hematopoietic cells, including enhancer-looping factor Ldb1 (refs 16, 42). Such cooperativity may explain why neither Scl nor Lmo2 is individually sufficient for reprogramming, and could underpin their synergistic action.

Though *Runx1* and *Bmi1* overexpression was dispensable for reprogramming, we found that the addition of these two factors atop *Scl* and *Lmo2* greatly enhanced the generation of long-term iHPs, especially in terms of CFU-S capacity and multi-month reconstitution capacity. As a target of Scl, *Runx1* is critical for generation of hematopoietic cells from hemogenic endothelium^11^,^12^,^50^. However, *Bmi1* is not thought to principally act in HSC specification during development, but rather has been accorded a role in the self-renewal of adult HSCs^18^,^38^, potentially enabling nascent HSCs to proliferate and self-renew to become repopulating cells. This is in accord with our findings that while *Bmi1* is not mandatory for iHP formation, its overexpression confers these cells with a ∼10-fold improvement for *in vivo* engraftment (Fig. 2a–c). Thus *Bmi1* seems to be a decisive factor that distinguishes the current iHP cells with multi-month engraftment potential from the cellular products of earlier protocols that lacked *Bmi1*, which we (Figs 3 and 4) and others^24^ observed to minimally engraft *in vivo*. However, our iHP cells still engraft less efficiently than whole BM cells (Fig. 2a–c, Supplementary Fig. 3a–c).

Though the mechanisms through which Bmi1 might act to endow iHP cells with enhanced self-renewal potential remain to be fully elucidated, Bmi1 is a core component of Polycomb Repressive Complex 1 (PRC1) which is thought to suppress transcription either through histone 2A mono-ubiquitination or nucleosome remodeling^51^. Though Bmi1 is thought to promote HSC self-renewal through suppression of *p16*^*Ink4a*^ and *p19*^*Arf*^ expression^18^, Bmi1 also physically interacts with Runx1 and indeed PRC1 co-occupies a wealth of target genes together with Runx1 and PRC1 recruitment to chromatin is even partially dependent on Runx1 (ref. 52). Given that both *Runx1* and *Bmi1* are simultaneously overexpressed to produce SLRB-iHPs, this might also underlie their synergistic action.

HoxB4 also provided a positive effect in iHP reprogramming separate from that of *Bmi1*. SLRH-iHP possessed both myeloid and erythroid-megakaryocyte potentials *in vitro* (Fig. 1 and Supplementary Fig. 2). Though SLRH-iHP engrafted even more efficiently in BM than SLRB-iHP at 16 wpt, their contribution was largely to the erythroid and megakaryocyte lineages (Fig. 3, Supplementary Fig. 4&5); by contrast, SLRB-iHPs could also form B-lymphoid cells and had more robust contribution to PB and SP. Ectopic expression of *HoxB4* has been well documented to confer *ex vivo* expansion and engraftment of adult HSC and progenitor cells^19^,^53^. Though high-level *HoxB4* ectopic expression confers human CD34^+^ cells with a growth advantage, it impairs their lymphomyeloid differentiation^54^. This is similar to what we observed for SLRH-iHP, with early multilineage contribution (at 2–5 wpt) but megakaryocyte-erythroid dominated long-term contribution (at 16 wpt). Given this genetic dosage sensitivity, whether careful modulation of HoxB4 expression levels can produce more robust long-term multilineage progenitors remains to be determined in the future.

**Figure 8:**
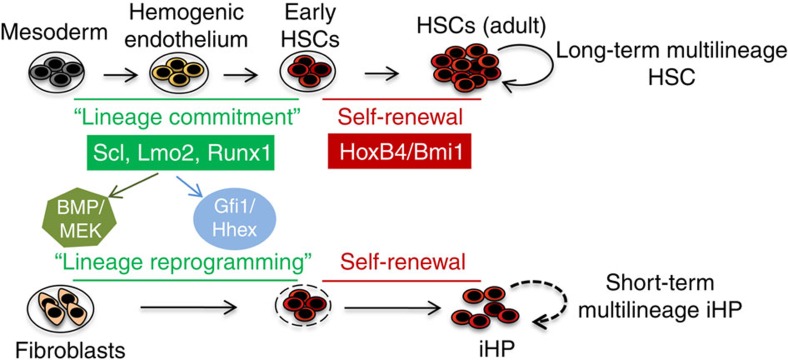
Model of hematopoietic reprogramming. Scl, Lmo2, Runx1 might act as ‘lineage commitment' regulators whereas Bmi1 or Hoxb4 might be ‘self-renewal' factors in HSC development. The hematopoietic reprograming activity of these transcription factors also jointly requires extracellular signals mediated through the BMP and MEK cascades. Finally, Lmo2 activates other hematopoietic transcription factors (for example, *Gfi1* and *Hhex*) to drive iHP reprogramming.

Altogether the sequential transcriptional code we have identified for hematopoietic reprogramming is evocative of some aspects of hematopoietic development: Scl and Lmo2 may be minimally sufficient to implement a hematopoietic fate (as evinced during embryogenesis), though we find that the numbers and engraftability of iHPs produced in this fashion are augmented by addition of Runx1 together with Bmi1, which might be reprising some of their roles as hematopoietic specification factors and self-renewal factors, respectively (Fig. 8). This type of a developmental transcriptional logic for lineage reprogramming may parallel what is seen for the conversion of adult mouse exocrine cells to β-cells, which involves three transcription factors (Pdx1, Ngn3 and MafA), which during embryogenesis serve to respectively induce pancreatic progenitors, then endocrine precursors and specifically β-cells^55^. Therefore ontogenic insights into how specific fates are programmed during development may reciprocally benefit how these fates can be reprogrammed from other lineages in artificial contexts.

Taken together our results suggest that a basic minimal combination of key hematopoietic transcriptional factors is critical and sufficient to rewrite the hematopoietic programme. We propose that these factors may be the equivalent of the ‘lineage commitment' factors that write and orchestrate lineage specification during embryonic hematopoiesis (Fig. 8). However, SLRB-iHP cells were not fully-fledged HSCs. Though iHPs could generate myeloid and B-lymphoid lineages *in vivo,* maximal reconstitution peaked at 5 weeks and often began declining thereafter, though donor contribution was observed until 16 weeks. In addition, it remains to be seen whether single iHP can generate both myeloid and B-lymphoid cell types, an issue that could be resolved through cellular barcoding strategies^56^. In approximating the phenotypic identity of our SLRB-iHPs to their rigorously-defined normal BM counterparts, we speculate that SLRB-iHPs are more akin to oligopotent progenitors^39^, as their self-renewal ability is limited by comparison with authentic long-term HSCs. A refined understanding of the regulators responsible for the differing self-renewal activity of LT-HSCs versus ST-HSCs/MPPs^57^ may be therefore key to identify additional reprogramming factors needed to induce fibroblasts into fully functional long-term HSCs. Therefore an expanded understanding of developmental regulators that control the maturation and self-renewal of HSCs is key to generate serially-transplantable iHPs, and we propose that the reprogramming system we describe here may be an ideal venue to validate and assay such regulators.

## Methods

### Plasmid construction

Mouse *Scl/Tal1* and *Gata2* and human *Lmo2*, *Runx1*, *HoxB4*, *Bmi1* and *Gfi1* were cloned into the pMX constitutively-expressed retroviral vector or the dox-inducible FuW-TetO lentiviral vector. Polycistronic insert mScl-F2A-hLmo2-T2A-hRunx1 (SLR) was cloned into pMX vectors using primers listed in Supplementary Table 2. Briefly, mScl-F2A and F2A-Lmo2-T2A were amplified from pMx-mScl and pMx-hLmo2, respectively. PCR products were gel purified and used as templates for amplification of mScl-F2A-Lmo2-T2A which was then cloned into pMx-hRunx1 to generate polycistronic construct pMX-mScl-F2A-hLmo2-T2A-hRunx1 (SLR). shRNA sequences of *Gfi1* and *Hhex* (see supplementary Table 2) were cloned into pLKO.1 TRC vector (http://www.addgene.org/tools/protocols/plko/).

### Cell culture and virus production

tdTomato^+^ MEFs were derived from E12.5−E13.5 embryos of the membrane-targeted tdTomato (mT) reporter mice strain (mT/mG, JAX mice stock No: 007676). Fibroblasts were prepared, cultured and transduced as described^4^. Fibroblasts were purified through removing of any cells that positive stained with a cocktail of hematopoietic makers by sorting (FACSAria llu SORP cell sorter, Becton Dickinson).

Mono antibodies (eBioscience) used for fibroblast purification are listed in the following: Sca1 FITC (catalogue no. 11-5981), CD150 APC (Catalogue no. 17-1501), CD48 APC (Catalogue no. 11-0481), c-kit APC (Catalogue no. 17-1172), CD41 APC (Catalogue no. 17-0411), AA4.1 APC (Catalogue no. 17-5892), CD45 APC (Catalogue no. 17-0451), Gr-1 APC (catalogue no. 17-5931), CD11b-APC (catalogue no. 17-0112), F4/80-APC (catalogue no. 17-4801), CD19 APC (catalogue no. 17-0193), B220 APC (catalogue no. 17-0452), Ter119 APC (Catalogue no. 17-5921), CD3e APC (catalogue no. 17-0031), CD4 APC (Catalogue no. 17-0041), CD8 APC (Catalogue no. 17-0081), CD31 APC (Catalogue no. 17-0311).

Hematopoietic cell-free fibroblasts transduced with hematopoietic factors were co-cultured with inactivated OP9 stromal cells and maintained in Iscove's modified Dulbecco's medium (IMDM) medium supplemented with 10% fetal bovine serum (FBS) and cytokine cocktail (SCF, IL3, TPO, FLT3L, 10 ng ml^−1^ each, R&D Systems) for observation of appearance of ‘cobblestone' colonies and generation of iHPs for FACS, molecular and functional analysis. Doxycycline (dox, 4 μg ml^−1^) were administrated during the reprogramming process if inducible factors were used.

### Colony-forming cell (CFC) assay

Methylcellulose colony-forming assay (CFC) was carried out using Methocult GF M3434 (Stem Cell Technologies). In all, 10^4^ suspension cells or sorted cells were seeded in 1 ml of Methocult GF M3434 in triplicate. Doxycycline (dox, 4 μg ml^−1^) were applied at the beginning of CFC assay for inducible factors. Colonies were scored at 12 days of culture based on standard morphological criteria and selectively validated by cytospin and staining.

### CFU-MK assay

*In vitro* Culture of Colony-Forming Unit- Megakaryocyte Assay (CFU-Mk) assay was done using MegaCult**-**C medium (Stem Cell Technologies) according to its technical manual (version 3.3.0). The cytokines used were TPO (50 ng ml^−1^), IL-3 (10 ng ml^−1^), IL-6 (20 ng ml^−1^) and IL-11 (50 ng ml^−1^). 50,000 cells (of total supernatant cells or sorted cells) were cultured in the MegaCult medium containing the cocktail of growth factors mentioned above in duplicates. Doxycycline (dox, 4 μg ml^−1^) were applied at the beginning of CFU-MK assay for inducible factors. By day 12–16, the CFU-Mks/CFU-mixs colonies were fixed in acetone, stained with AchE and recorded according to protocol from kit.

### Cytospin and benzidine-Wright–Giemsa staining

iHP cells or single colonies (5 × 10^4^) from in CFC assay were suspended in 100 μl of 10% FBS in PBS and cytospined to the slides. The slides were fixed with cold methanol for 2 min and allowed to dry for 1 h. Then slides were stained using DAB tablets (3, 3′-Diaminobenzidine tetrahydrochloride, Sigma), followed by stained with Wright–Giemsa solution (Sigma) according to the instruction of products. The slides were allowed to dry overnight, mounted with mounting medium and photographed under microscope (Leica DM LB2).

### Phagocytosis assay

35–50 dpi suspension cells were assayed using Cayman's Phagocytosis Assay Kit (Cayman chemical) according to instructions included in the kit. Briefly, 10^6^ cells were cultured with latex beads-Rabbit IgG-FITC in a CO_2_ incubator at 37 °C in dark for 24 hrs. The washed cells were stained with hematopoietic markers (eBioscience, Gr-1-APC, CD11b-APC, CD45-PE (catalogue no. 12-0451), c-kit-PE (catalogue no. 12-1171) and CD41-PE (catalogue no.12-0411)) for FACS analysis or fluorescence microscopy (zeiss observer.D1).

### Flow cytometry

Flow cytometry was performed using FACSAria llu SORP cell sorter (Becton Dickinson) and analysis was performed using BD FACSDiva software. Compensation was performed with single stained controls and gating was performed on fluorochrome minus one controls, unstained controls and isotype controls.

Mono antibodies (eBioscience) used in hematopoietic lineages analysis were listed below. CD45 eFluor 450 (catalogue no. 48-0451), CD45.2 eFluor 450 (catalogue no. 48-0454), Gr-1 APC, Gr-1 FITC (catalogue no. 11-5931), CD11b-APC, F4/80-APC, CD71 eFluor 450 (catalogue no. 48-0711), Ter119 APC, CD41 eFluor 450 (catalogue no. 48-0411), CD61 FITC (catalogue no. 11-0611), CD42d APC (catalogue no. 17-0421), CD3e FITC (catalogue no. 11-0031), CD4 FITC (catalogue no. 11-0041), CD8a FITC (11-0081), CD19 APC, CD19 FITC (Catalogue no.11-0193), B220 APC.

### Immunofluorescence immunostaining

Transduced fibroblasts (27–30 dpi) with obvious ‘cobblestones' in 24-well plates were fixed with 4% PFA, blocked with 10% normal rat serum (eBioscience, catalogue no. 24-5555) and then stained with CD41PE (eBioscience, catalogue no. 12-0411) or c-kit FITC (catalogue no. 17-1172) and photographed under microscope (zeiss observer.D1).

### CFU-S assay and engraftment assay

For *in vivo* Colony-Forming Unit-Spleen (CFU-S), 6–8 weeks old female SCID mice (C.B-17/IcrHanHsd-Prkdc^scid^) and C57BL/6J mice (Jax mice) were lethally irradiated with 4 and 7.5 Gy respectively 4 h before transplantation. Two or five million of loosely attached and suspension cells (30–50 dpi, tdTomato^+^) or one million of sorted kit^+^ cells from 30–50 dpi tdTomato MEF were transplanted by tail vein injection. Spleens were dissected from 12 dpt mice and tdTomato^+^ cells/nodules in the spleen were visualized, photographed or counted under microscope (Leica M205 FA). CFU-S12 were enumerated after tdTomato^+^ spleens were fixed in carnoy's fixative and photographed. Frequencies of CFU-12 or tdTomato^+^ nodules are represented as nodules from one side of the spleen per spleen. Donor (tdTomato^+^) cells contribution in SP, PB and BM were evaluated by FACS analysis at 12–14 dpt.

For long-term engraftment experiment, five million of loosely attached and suspension cells (tdTomato^+^, 30–50 dpi) were transplanted by tail vein injection in sub-lethally irradiated 6–8 weeks old female immunocompromised strains (NSG (NOD.Cg-Prkdc^scid^ Il2rgtm1Wjl/SzJ, Jackson laboratory) or Nod-scid mice (NOD/MrkBomTac-Prkdc^scid^), 3 Gy) and 6–8 weeks old female immunocompetent strains (6–8 weeks old female C57Bl/6J mice, 6 Gy). Transplanted mice were killed at 5 wpt and 16 wpt to analyse the donor tdTomato^+^ cells contribution in PB, SP and BM by flow cytometry (FACSAria llu SORP cell sorter, Becton Dickinson). A total of 16 wpt BM cells were placed for CFC assay and transplanted secondarily into irradiated Nod-scid mice (∼7–9 million cells per mouse). Animal experiments were performed under the guidelines set by the Institutional Animal Care and Use Committee (IACUC).

### V(D)J recombination and transgene integration assays

Triple positive (tdTomato^+^/CD45.2^+^/ (CD19+B220)^+^) SLRB-iHP B cells (polycistronic SLR was used) in 16 wpt combined spleen cells were sorted by flow cytometry (FACSAria llu SORP cell sorter, Becton Dickinson). Control T or B cells were sorted from spleen of C57BL/6 mice. Single cells were manually picked under microscope from the respective sorted samples. Single control MEF cells were SLRB infected tdTomato^+^ MEF (14 dpi, polycistronic SLR was used). Total spleen genomic DNA (gDNA) were extracted using TRIzol reagent (Ambion). Whole-genome amplification (WGA) of single cell was performed using the REPLI-g Single Cell kit (Qiagen). The amplified DNA was diluted (1: 100) for PCR assay. WGA gDNA was evaluated and genotyped with tdTomato primer before used for VDJ recombination analysis (Supplementary Table 1, http://jaxmice.jax.org/strain/007676.html). V(D)J recombination assay by PCR was performed using primers listed in Supplementary Table 2 as previously described^58^,^59^. The V(D)J recombination PCR products were randomly selected and confirmed by the southern blot using internal oligonucleotide probes listed in Supplementary Table 2. Probes were labelled with DIG labelled oligonucleotide Tailing Kit, 2nd generation (Roche). The blots were scanned with Image lab software (Bio-rad). The PCR reaction was carried out using DreamTaq Green PCR Master Mix (Thermo Scientific). Primers sequences for transgene integration are provided in Supplementary Table 2.

### qPCR and microarray

Total RNA was isolated using RNeasy mini/micro kit with on-column DNaseI digestion of genomic DNA (Qiagen). cDNA synthesis and Quantitative PCR (qPCR) were performed using superscript III (Invitrogen) and SYBR Green (Applied Biosystems; qPCR primer sequences are provided in Supplementary Table 2).

For whole-genome expression profiling, microarray analysis was performed on samples: tdTomato-MEF (0 dpi, empty vector infected), SLHR infected tdTomato-MEF/intermediates (4 dpi, 14 dpi), Kit^+^/Kit^−^/CD45^+^/CD45^−^ cells of 26 dpi tdTomato-iHP cells and Kit^+^/Kit^−^/CD45^+^/CD45^−^ cells from tdTomato BM cells. SLHR factors were singly delivered in individual pMX vectors. Total RNA were labelled with Cy3, and hybridized to MouseRef-8v2 Beadchip (Illumina) according to the manufacturer's protocol. Arrays were scanned with Beadchip Reader (Illumina).

### ChIP-seq library preparation

SLHR infected tdTomato^+^ MEF (4 dpi) were used for ChIP analysis. SLHR factors were singly delivered in individual pMX vectors. The genomic DNA obtained from Lmo2 ChIP (anti- hLmo2, AF2726, R&D Systems) experiments were used to prepare paired end sequencing libraries using TruSeq ChIP Sample Preparation Kit (Illumina, IP-202-1012) as per manufacturer's instructions.

### Microarray data analysis

Raw arrays were normalized with the quantile normalization algorithm using the bioconductor package lumi (http://www.bioconductor.org/packages/release/bioc/html/lumi.html)^60^. Cluster analysis of the normalized reads was performed using Pvclust (http://www.is.titech.ac.jp/~shimo/prog/pvclust/) which was installed in the R project (http://www.r-project.org/). The D0 (0 dpi), D4 (4 dpi), iHP/BM CD45^+^ and Kit^+^ microarray chips have undergone quantile normalization using IlluminaNormalizer module on GenePattern platform (http://genepattern.broadinstitute.org/; http://www.broadinstitute.org/cancer/software/genepattern/)^61^. The normalized values were transformed to Log2. Published microarray expression data were downloaded from (http://daleystem.hms.harvard.edu/)^40^. The datasets from the two studies were merged and adjusted for systematic microarray data biases using Distance Weighted Discrimination^62^. Hierarchical clustering of the samples from the two studies have been performed using ‘hclust' function in R. Heatmaps were generated using gplots package (http://cran.r-project.org/web/packages/gplots/index.html).

### Identifying target genes regulated by Lmo2

To identify the genes that are regulated by Lmo2, we first determined the genes that show significant change in gene expression at 4 dpi and 14 dpi cells. At 4 dpi cells, genes that show a minimum of 20% change (up or down) with a *P* value of 0.1 or below, were considered for further analysis. At 14 dpi, genes that show 2 or more fold change (up or down) with a *P* value of 0.1 or below were considered for further analysis.

Then, to consider a gene to be bound by 4 dpi Lmo2, there should be at least one peak of 4 dpi Lmo2 that could be located anywhere in a window, starting from 35 K upstream of the TSS of the genes and ending at the TTS of the gene.

Lists of genes bound by 4 dpi Lmo2 were correlated with lists of genes that show significant change in gene expression. These correlated lists of genes were annotated using metascape.

### Dynamic gene expression analysis

The dynamic gene expression analysis was performed using GEDI v2.1 (ref. 41) with a grid size of 80x84.

### ChIP-Seq data analysis

Quality control analysis of the raw reads was performed using FastQC (http://www.bioinformatics.babraham.ac.uk/projects/fastqc/). Reads were mapped to mm9 genome using STAR aligner software^63^. Reads mapping to more than one locus and/or having more than three mismatches were filtered out. Mapped reads were subjected to ngsplot tool^64^ to generate the distance to TSS plots and enrichment heatmaps. The significant regions of H3K4me3, H3K37ac and H3K27me3 binding were detected using HOMER^65^. HOMER was used to normalize the mapped reads and convert the mapped files to bedGraph format to visualise them in the UCSC genome browser (http://genome.ucsc.edu/)^66^ using mm9 as the genome assembly.

The published histone raw data used have the accession numbers: GSM1246686, GSM1246689 and GSM1246690 for MEF H3K4me3, H3K27ac and H3K27me3 ChIP-Seq samples respectively^44^ and were downloaded from the Gene Expression Omnibus, NCBI database^67^. The peaks were annotated using annotatePeaks.pl script of HOMER^65^. Correlation of those genes with microarray data were performed using the online Venn Digram Generation tool (http://bioinformatics.psb.ugent.be/webtools/Venn/).

### Gene ontology (GO) analysis

GO analysis was performed using BiNGO app^68^ which is installed in Cytoscape v3.2.1 (ref. 69). The GO terms (OBO v1.2) and the MGI Mouse GO annotations were downloaded from the Gene Ontology Network website (http://www.geneontology.org/) on 1st of July, 2015. GO terms with *P* values>0.01 were filtered out.

### Pathway mapping

Pathway mapping was performed using DAVID web resource (http://david.abcc.ncifcrf.gov/). Genes, which are upregulated at 14 dpi cells were uploaded to DAVID. The KEGG pathways enriched in these genes were identified. The Pathway map was generated using DAVID.

### Data availability

The authors declare that all data supporting the findings of this study are available within the article and its Supplementary Information files. Microarray and ChIP-Seq data have been deposited in GEO database under accession code GSE86198.

## Additional information

**How to cite this article:** Cheng, H. *et al*. Reprogramming mouse fibroblasts into engraftable myeloerythroid and lymphoid progenitors. *Nat. Commun.* **7**, 13396 doi: 10.1038/ncomms13396 (2016).

**Publisher's note:** Springer Nature remains neutral with regard to jurisdictional claims in published maps and institutional affiliations.

## Supplementary Material

Supplementary Information Supplementary Figures 1-10, Supplementary Tables 1-2

Supplementary Data 1 4dpi Lmo2 bound-genes. List of genes that bound by Lmo2 at 4dpi in SLHR infected MEF.

Supplementary Data 2 4dpi Lmo2-bound-genes that are up regulated at 4dpi. List of genes that are bound by Lmo2 at 4dpi and upregulated in expression at 4dpi in SLHR infected MEF.

Supplementary Data 3 4dpi Lmo2-bound-genes that are down regulated at 4dpi. List of genes that are bound by Lmo2 at 4dpi and downregulated in expression at 4dpi in SLHR infected MEF

Supplementary Data 4 4dpi Lmo2-bound-genes that are up regulated at 14dpi. List of genes that are bound by Lmo2 at 4dpi and upregulated in expression at 14dpi in SLHR infected MEF

Supplementary Data 5 4dpi Lmo2-bound-genes that are down regulated at 14dpi. List of genes that are bound by Lmo2 at 4dpi and downregulated in expression at 14dpi in SLHR infected MEF
