# Supplementary material for: Reprogramming mouse fibroblasts into engraftable myeloerythroid and lymphoid progenitors
Source: Nat Commun. 2016 Nov 21;7:13396. doi: 10.1038/ncomms13396 (PMC5121332; doi:10.1038/ncomms13396)
Supplement: Supplementary Information — Supplementary Figures 1-10, Supplementary Tables 1-2 [file ncomms13396-s1.pdf]

Supplementary Figures and Tables

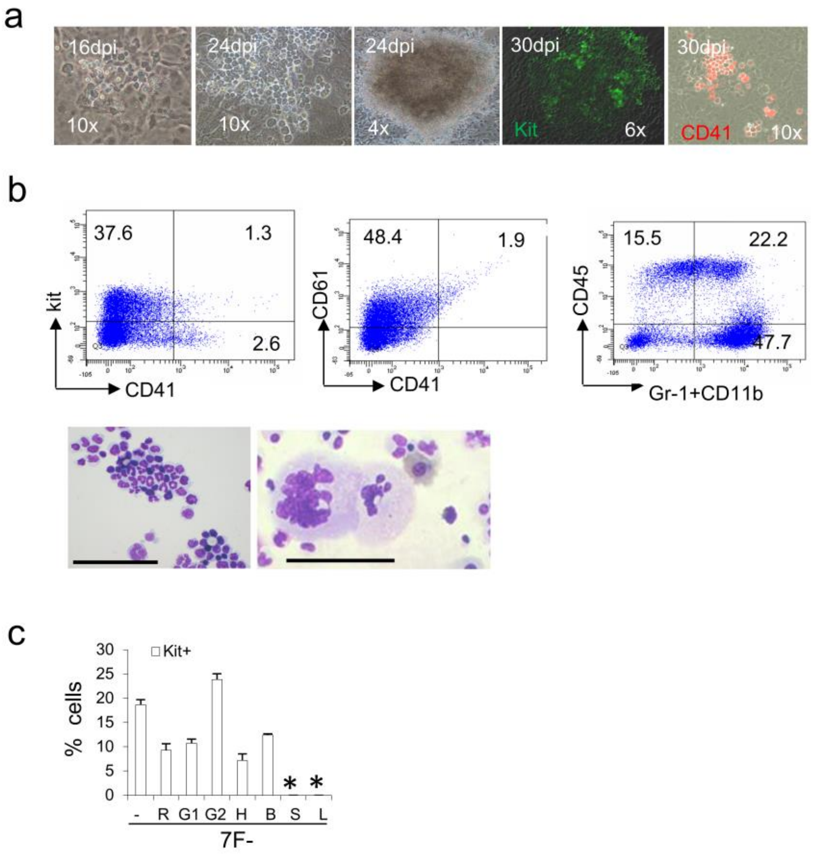

Supplementary Figure 1: Induced hematopoietic progenitors (iHP) in *p53*$^{-/-}$ MEF

(a) 7F-induced 'cobblestone' colonies as viewed under bright field and 'cobblestone' colonies stained positively with stem/progenitor markers: Kit and CD41. These images are representatives of three independent experiments in *p53*$^{-/-}$ background.

(**b**) FACS analysis of 35dpi 7F-induced suspension cells with hematopoietic markers and morphological characterization of these cells with Wright-Giemsa-Bezidine staining. Scale bar: 100µm. These are representatives of three independent experiment in *p53*$^{-/-}$ background.

(**c**) Removal of S or L from cocktail (7F) failed to generate any hematopoietic-like round cells. Removal of other factors (R, G1, G2, H and B) individually permitted the production of Kit^{+} cells. Data are shown as mean ± SD (Standard deviation) of biological triplicates in *p53*$^{-/-}$ background.

These data are generated from factors in pMX vector.

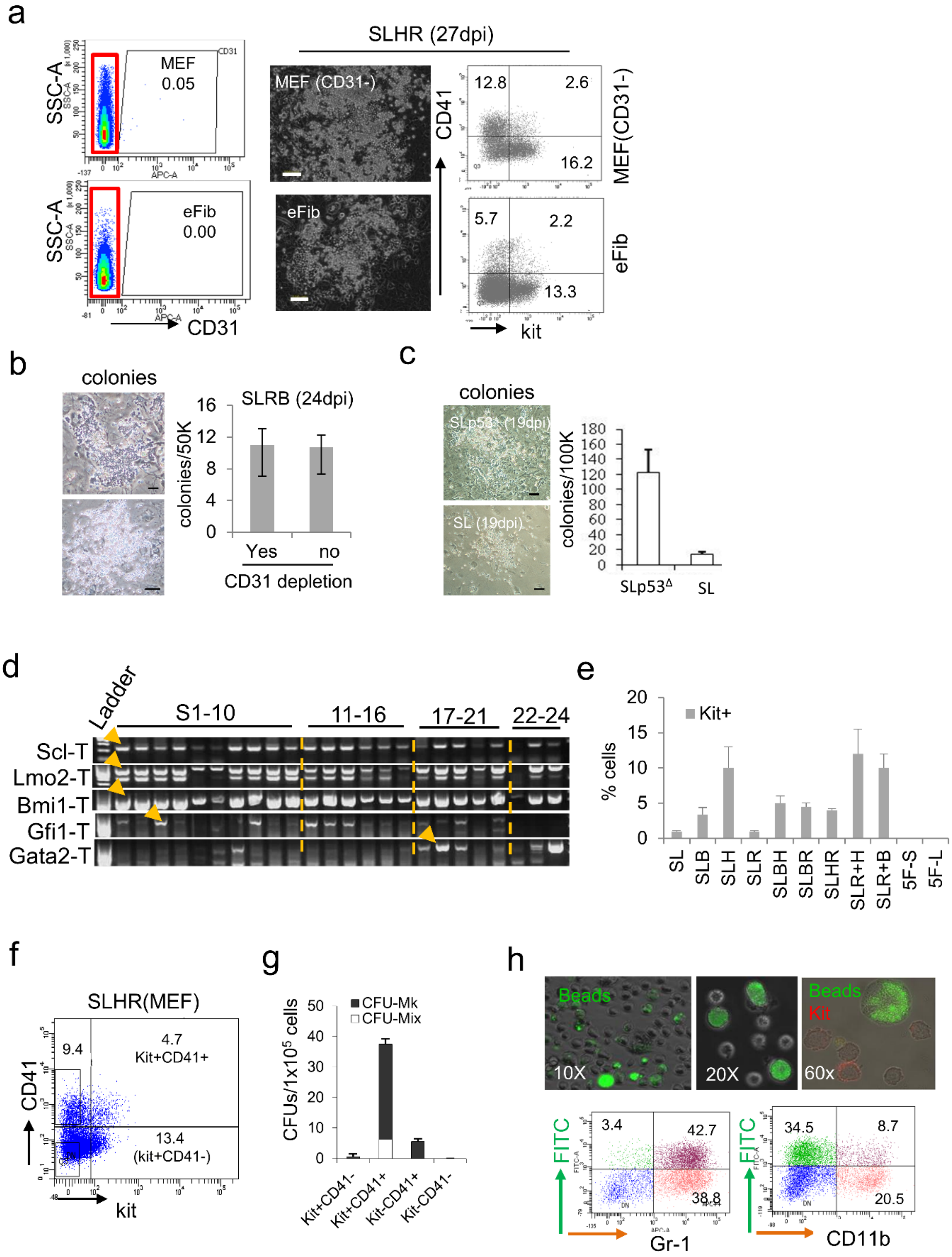
a
SLHR (27dpi)
SSC-A
MEF
0.05
CD31
eFib
0.00
APC-A
MEF (CD31-)
eFib
CD41
12.8
2.6
16.2
5.7
2.2
13.3
MEF(CD31-)
eFib
kit
b
colonies
SLRB (24dpi)
colonies/50K
16
12
8
4
0
Yes
no
CD31 depletion
c
colonies
SLp53Δ (19dpi)
SL (19dpi)
colonies/100K
180
160
140
120
100
80
60
40
20
0
SLp53Δ
SL
d
Ladder
S1-10
11-16
17-21
22-24
Scl-T
Lmo2-T
Bmi1-T
Gfi1-T
Gata2-T
e
Kit+
% cells
20
15
10
5
0
SL
SLB
SLH
SLR
SLBH
SLBR
SLHR
SLR+H
SLR+B
5F-S
5F-L
f
SLHR(MEF)
CD41
9.4
4.7
Kit+CD41+
13.4
(kit+CD41-)
kit
g
CFU-Mk
CFU-Mix
CFUs/1x10^5 cells
50
40
30
20
10
0
Kit+CD41-
Kit+CD41+
Kit-CD41+
Kit-CD41-
h
Beads
10X
20X
Beads
Kit
60x
FITC
3.4
42.7
38.8
Gr-1
FITC
34.5
8.7
20.5
CD11b

Supplementary Figure 2: iHP in wild type background

(**a**) iHP reprogramming from CD31 depleted MEF and eFib (ear Fibroblast). FACS showed residual CD31$^+$ cells in MEF but no CD31$^+$ cells in eFib. Hematopoietic colonies (cobblestone/round cells) and CD41/Kit cells were induced by SLHR from CD31-depleted MEF and eFib. Factors were in pMX vector and were delivered individually. These are representatives of four independent experiments.

(**b**) SLRB induced hematopoietic colonies and frequencies of these colonies from CD31-depleted MEF and CD31-undepleted MEF. Factors were in pMX vector. Polycistronic SLR was used. These data are from four independent experiments.

(**c**) Representative hematopoietic colonies in SLp53$^{\Delta}$ and SL infected MEF and frequencies of these colonies. S, L, p53$^{\Delta}$ stand for Scl, Lmo2 and p53DD in pMX vector. These data are from four independent experiments.

(**d**) Tran-gene integration in single CFU-colonies derived from FuW-TetO-7F-iHP. S1-10, S11-16, S17-21 and S22-24 represent individual single colonies from four independent experiments.

(**e**) H or B enhances SL on production of Kit$^+$ cells. 27dpi suspension cells were analyzed by FACS. 5F: S, L, R, H and B (in pMX vector). SLHR and SLBR denote factors delivered individually. SLR+H and SLR+B stand for SLR in one polycistronic construct (polycistronic SLR) were delivered, together with H or B was singly delivered. Data are shown as mean ± SD of biological triplicate.

f) Representative FACS of SLHR induced suspension cells (27dpi) with hematopoietic markers: Kit and CD41. Percentage of different population of cells are shown on the plot. Factors were in FuW-tetO vectors. This is representative of three independent experiments.

(**g**) Frequencies of CFU-Mk and CFU-mix colonies derived from CFU-Mk assay (collagen-based) using 27dpi SLHR-iHP cells. Colonies derived from different sorted subsets were shown as mean ± SD of biological triplicate. Factors were in FuW-TetO vector.

(**h**) Phagocytosis of FITC-latex beads of SLHR induced suspension cells. 50dpi SLHR induced suspension cells (>1 million) were used for analysis. Factors in pMX vector were individually delivered. These data are representative of three independent experiments.

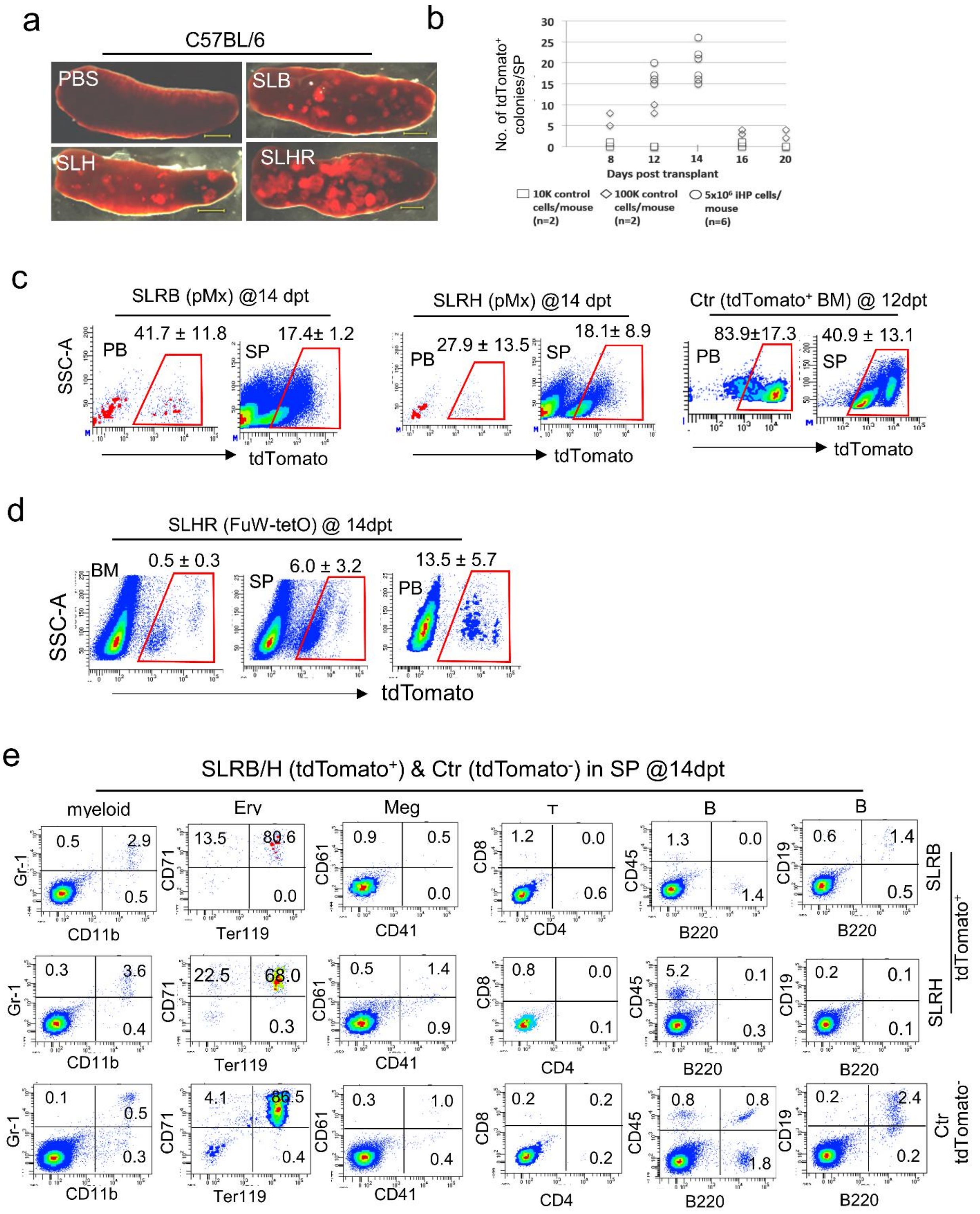

Supplementary Figure 3: *In vivo* engraftment at 12-14 days post transplantation

(**a**) tdTomato$^+$ nodules or CFU-S formation in the spleen (SP) of tdTomato$^+$-iHP cells transplanted C57Bl/6 mice (12dpt, days post transplantation). For SLH-, SLB-, and SLHR-iHP cells: 5x10^6 cells per mouse were transplanted. Factors in pMX vector were

individually delivered. Scale bar: 2mm. These are representative of three independent experiments.

(**b**) Summary of frequency of tdTomato$^+$ nodules in the SP of SLHR-iHP cells transplanted C57Bl/6 mice (12dpt). tdTomato$^+$ nodules are counted from one side of the spleen. These data generated from one single experiment.

(**c**) tdTomato$^+$ cells detected in the SP and peripheral blood (PB) of SLRB/H-iHP cells (tdTomato$^+$) and control BM cells (Ctr, tdTomato$^+$) transplanted SCID mice (12-14 dpt). For SLRB/H-iHP cells: $2x10^6$ cells per mouse were transplanted; for Ctr BM cells (tdTomato$^+$), $2x10^5$ cells per mouse were transplanted. Percentage of tdTomato$^+$ cells are shown as mean ± SD (mice n=6 for each type of injected cells). Factors were in pMX and polycistronic SLR was used. These are from three independent experiments.

(d) tdTomato$^+$ cells detected in the BM, SP and PB of FuW-TetO-SLHR-iHP cells ($4x10^6$ cells, tdTomato$^+$) transplanted SCID mice (14 dpt). Dox were administrated during reprogramming (*in vitro*) and stopped after transplantation (no dox in *in vivo*). Percentage of tdTomato$^+$ cells are shown as mean ± SD (mice n=5 for each type of cells). These are from two independent experiments.

(**e**) Multilineages contribution of SLRB/H-iHP cells (tdTomato$^+$) in SP at 12-14 dpt. SCID mice were transplanted. Different lineage cells (tdTomato$^+$) are stained with markers as shown on the plots. tdTomato$^-$ recipient cells were presented as control (Ctr). Percentages of cells were presented on the plots. Factors were in pMX and polycistronic SLR was used. These are representatives of three independent experiments (mice n=6 for each type of cells).

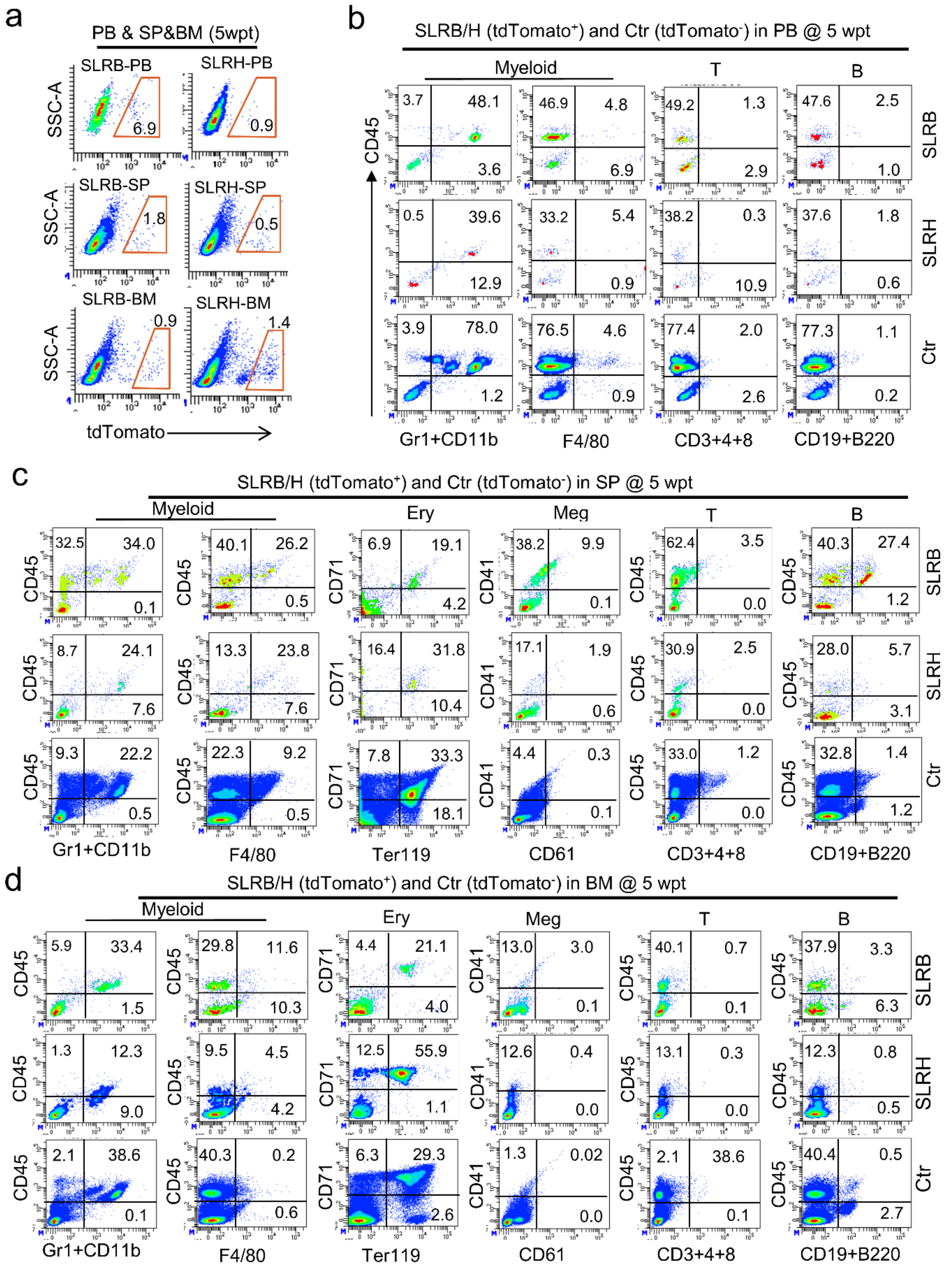
a
PB & SP&BM (5wpt)
SLRB-PB
SLRH-PB
SLRB-SP
SLRH-SP
SLRB-BM
SLRH-BM
SSC-A
tdTomato
b
SLRB/H (tdTomato+) and Ctr (tdTomato-) in PB @ 5 wpt
Myeloid
T
B
SLRB
SLRH
Ctr
CD45
Gr1+CD11b
F4/80
CD3+4+8
CD19+B220
c
SLRB/H (tdTomato+) and Ctr (tdTomato-) in SP @ 5 wpt
Myeloid
Ery
Meg
T
B
CD45
CD71
CD41
Gr1+CD11b
F4/80
Ter119
CD61
CD3+4+8
CD19+B220
SLRB
SLRH
Ctr
d
SLRB/H (tdTomato+) and Ctr (tdTomato-) in BM @ 5 wpt
Myeloid
Ery
Meg
T
B
CD45
CD71
CD41
Gr1+CD11b
F4/80
Ter119
CD61
CD3+4+8
CD19+B220
SLRB
SLRH
Ctr

Supplementary Figure 4: SLRB/SLRH-iHP cells engraft at 5 weeks post transplantation (wpt)

(**a**) SLRH/B-iHP cells (tdTomato+) engraft in PB, SP and BM (bone marrow) at 5 wpt. Polycistronic SLR was used. Mice analyzed n=5 for each type of cells. These are representative of three independent experiments

(**b**) Lineages contribution of SLRB/H-iHP cells (tdTomato^{+}) in PB at 5 wpt. Myeloid and lymphoid markers and percentages of cells were shown on the plots. Recipient PB cells (Ctr, tdTomato^{-}) were presented as ctr. Mice analyzed n=5 for each type of cells. Polycistronic SLR factors are used. These are representative of three independent experiments.

(**c**) Multilineages contribution of SLRB/H-iHP cells (tdTomato^{+}) in SP at 5 wpt. Different lineage cells (tdTomato^{+}) are stained with markers as shown on the plots. tdTomato^{-} recipient cells were as control (Ctr). Mice analyzed n=5 for each type of cells. Polycistronic SLR was used. These are representative of three independent experiments.

(**d**) Multilineages contribution in the BM of SLRB/H-iHP cells (tdTomato^{+}) at 5 wpt. Mice analyzed n=5 for each type of cells. Polycistronic SLR was used. These are representative of three independent experiments.

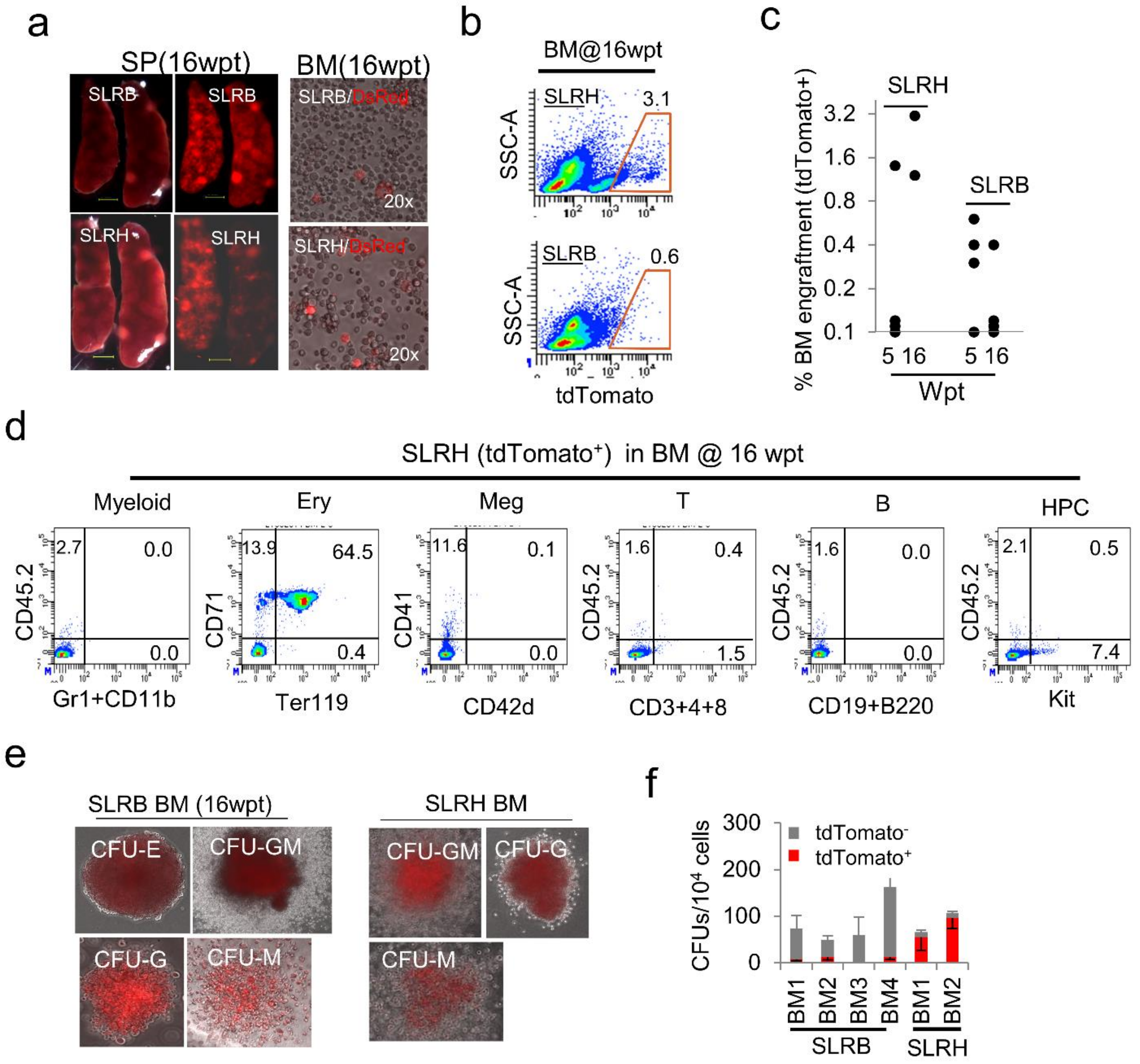

Supplementary Figure 5: SLRB/SLRH-iHP cells engraft up to 16wpt *in vivo*

(**a**) Representative pictures showing SLRH/B-iHP (tdTomato^{+}) cells engraft in 16 wpt SP and BM. Polycistronic SLR was used. These are representative of three independent experiments.

(**b**) SLRH/B-iHP (tdTomato^{+}) cells engraft in BM at 16 wpt. Polycistronic SLR was used. These are representative of three independent experiments.

(**c**) Summary of SLRH/B-iHP (tdTomato+) cells engraftment in BM at 5 wpt (mice n=5 for each type of cells) and 16wpt (mice n=3 for SLRH iHP, n=9 for SLRB-iHP). Only

tdTomato$^+$ cells ≥ 0.1% were shown in the plot. Polycistronic SLR was used. These data are from three independent experiments.

(**d**) SLRH-iHP (tdTomato+) cells in 16wpt BM contributed predominantly to CD71$^+$Ter119$^+$ cells, followed by CD41$^+$ cells and kit$^+$ cells, with few CD45$^+$ cells, and no lymphoid positive cells. Polycistronic SLR was used. These are representative of two experiments.

(**e**) Representative tdTomato$^+$ CFU-E, -GM or -G/M colonies derived from 16 wpt BM. Total BM cells (of SLRB/H transplanted mice) were used for CFC assay. Polycistronic SLR was used. These are representative of two experiments.

(**f**) Frequency of tdTomato$^+$/TdTomato$^-$ colonies observed in CFC assay of 16 wpt total BM cells. BM1-4 denote BM cells from mice 1-4. Low no of SLRB-iHP derived tdTomato$^+$ colonies were detected in 16 wpt BM. SLRH-iHP cells derived tdTomato$^+$ colonies were the major colonies in their respective BM. Data were mean ± SD of biological triplicate. These is from single experiment

a b

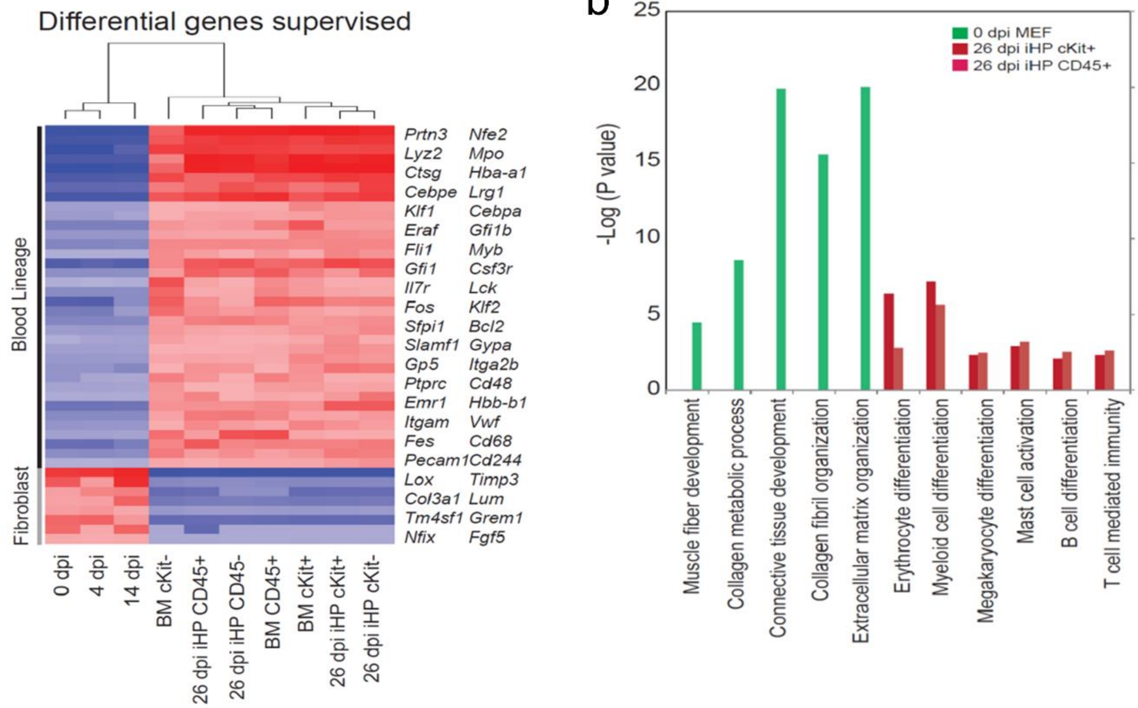

c

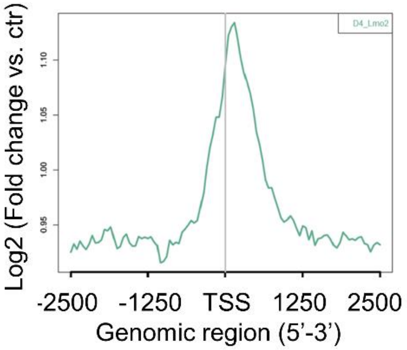

d

Number of Input Reads	19022071
Number of Uniquely mapped Reads	15735516
% of Uniquely Mapped Reads	82.72%

e

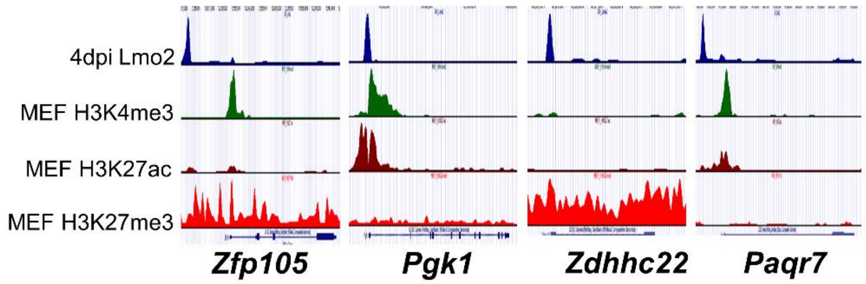

Supplementary Figure 6: Microarray and ChIP-seq analysis of iHP and intermediates

(**a**) Heatmap generated from supervised differential genes expression analysis. iHP showed increased expression of genes that are characteristic of blood lineage compared to 0dpi cells, 4dpi cells and 14dpi cells. 14dpi cells show minimal expression of genes characteristic of blood progenitor cells and high expression of genes characteristic of fibroblast lineage instead. Data drawn from the average of two independent experiments.

(**b**) Highly enriched genes in microarray (Fold change > 2, FDR < 0.01) were subjected to gene ontology analysis using BiNGO. 0dpi cells show high enrichment of fibroblasts' functions, represented by green bars, whereas reprogrammed c-Kit$^+$ and CD45$^+$ show high enrichment of blood functions, represented by red and orange bars respectively. This clearly indicates the switch in the cells' fate.

(**c**) Binding profile of 4dpi Lmo2 ChIP-Seq shows a high enrichment of mapped reads around the TSS

(**d**) The mapping statistics of 4dpi Lmo2 shows a high percentage of uniquely mapped reads. This indicates that the library is reliable for analysis

(**e**) UCSC screenshots showing examples of Lmo2 bound genes at different chromatin states

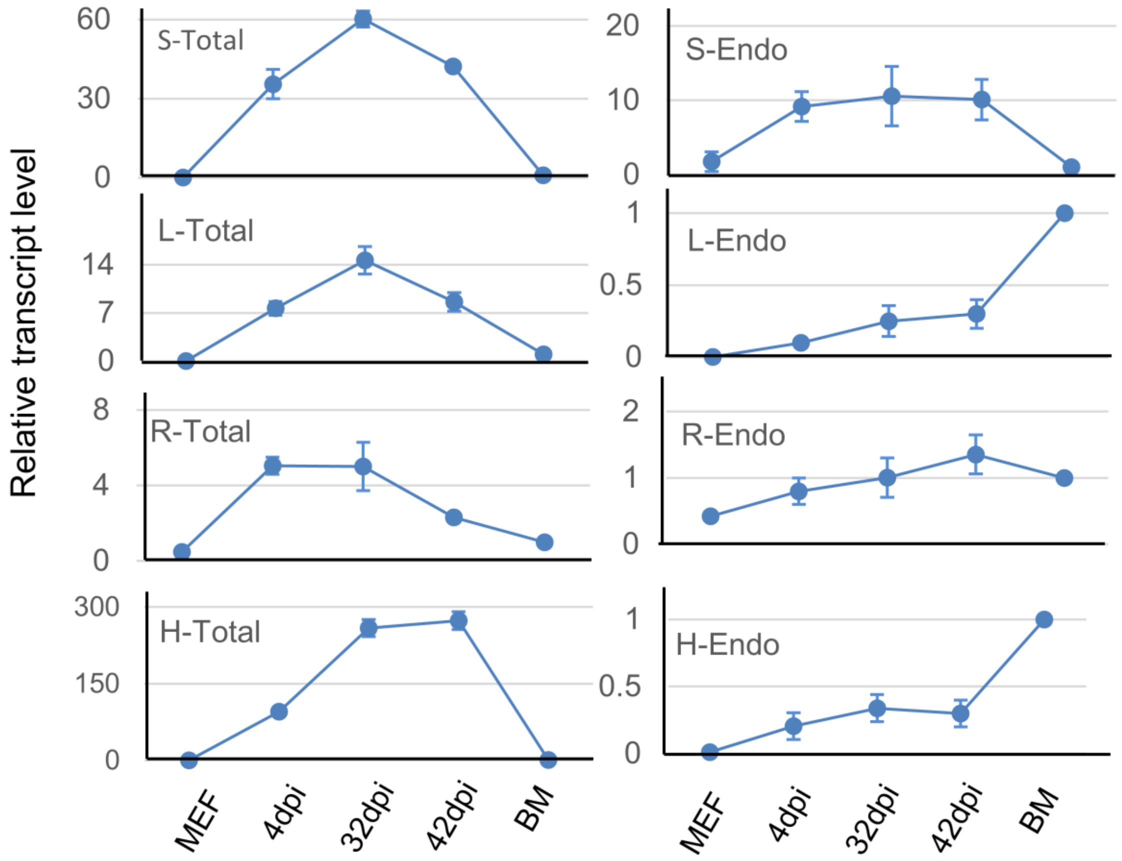

Supplementary Figure 7: Activation of endogenous factors during iHP reprogramming

Total (including transgenes and endogenous genes) and endogenous (Endo) expression of Scl (S), Lmo2 (L), Runx1 (R) and HoxB4 (H) were analyzed at early stage (4dpi) and late stages (32 dpi and 42 dpi suspension cells). MEF and Bone marrow (BM, Kit+) cells were used as controls. Factors (S, L, R and H) were in pMx vector. Data were as mean ± SD of technical triplicate. These are representative of two experiments.

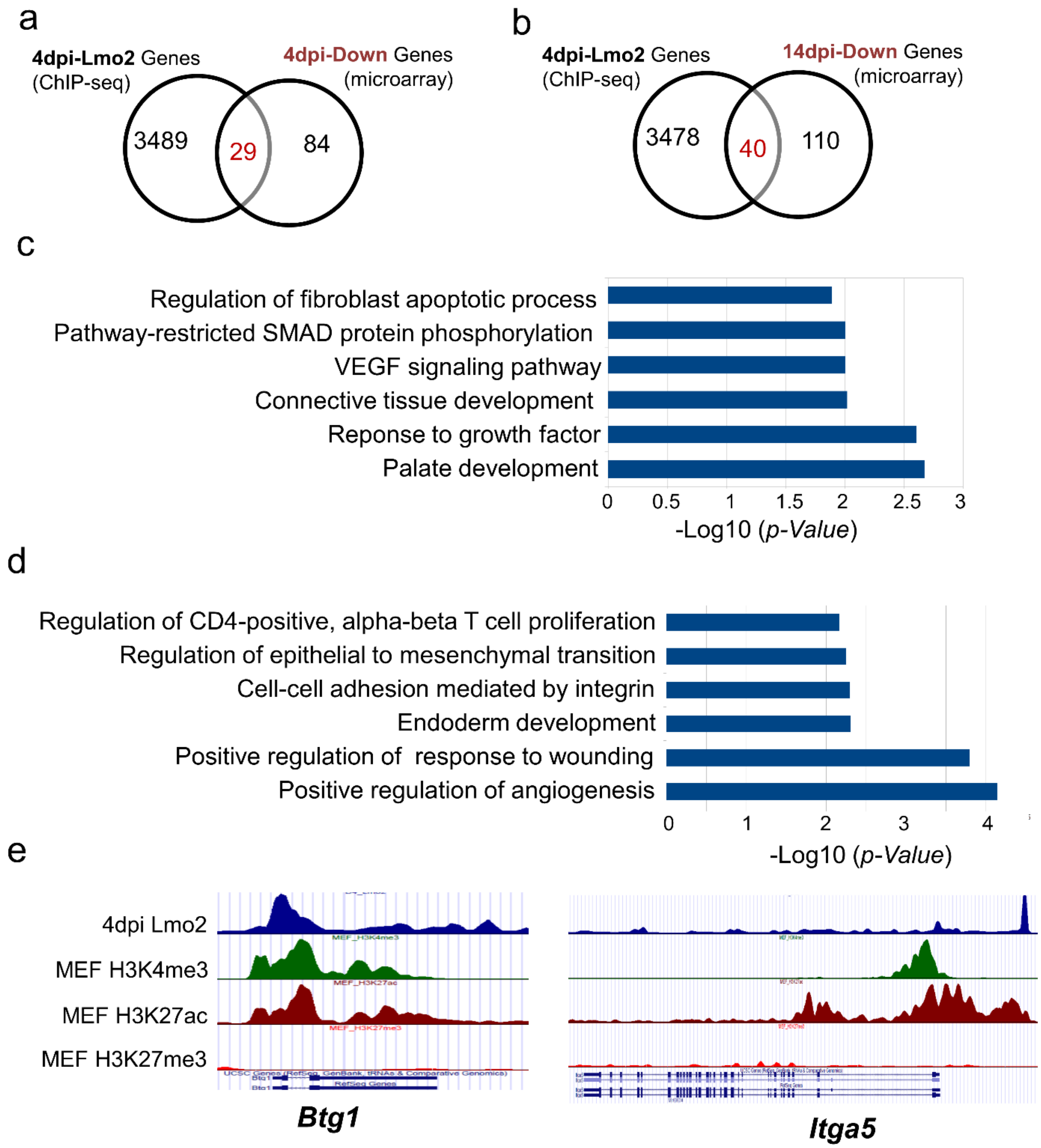

Supplementary Figure 8: Intersectional analysis of 4dpi Lmo2-bound genes and 4dpi /14dpi downregulated genes

(**a**) Venn diagram demonstrating the numbers of 4dpi Lmo2-bound genes that are downregulated at 4 dpi cells.

(**b**) Venn diagram demonstrating the numbers of 4dpi Lmo2-bound genes that are downregulated at 14 dpi cells.

(**c**) GO analysis of 29 genes that 4dpi Lmo2 bound and downregulated at 4dpi
(**d**) GO analysis of 40 genes that 4dpi Lmo2 bound and downregulated at 14dpi

(**e**) UCSC screenshots demonstrating the binding of 4dpi Lmo2 on promoter of *Btg1* (4dpi downregulated) and *Itga5* (14 dpi downregulated). These are representatives from a single experiment.

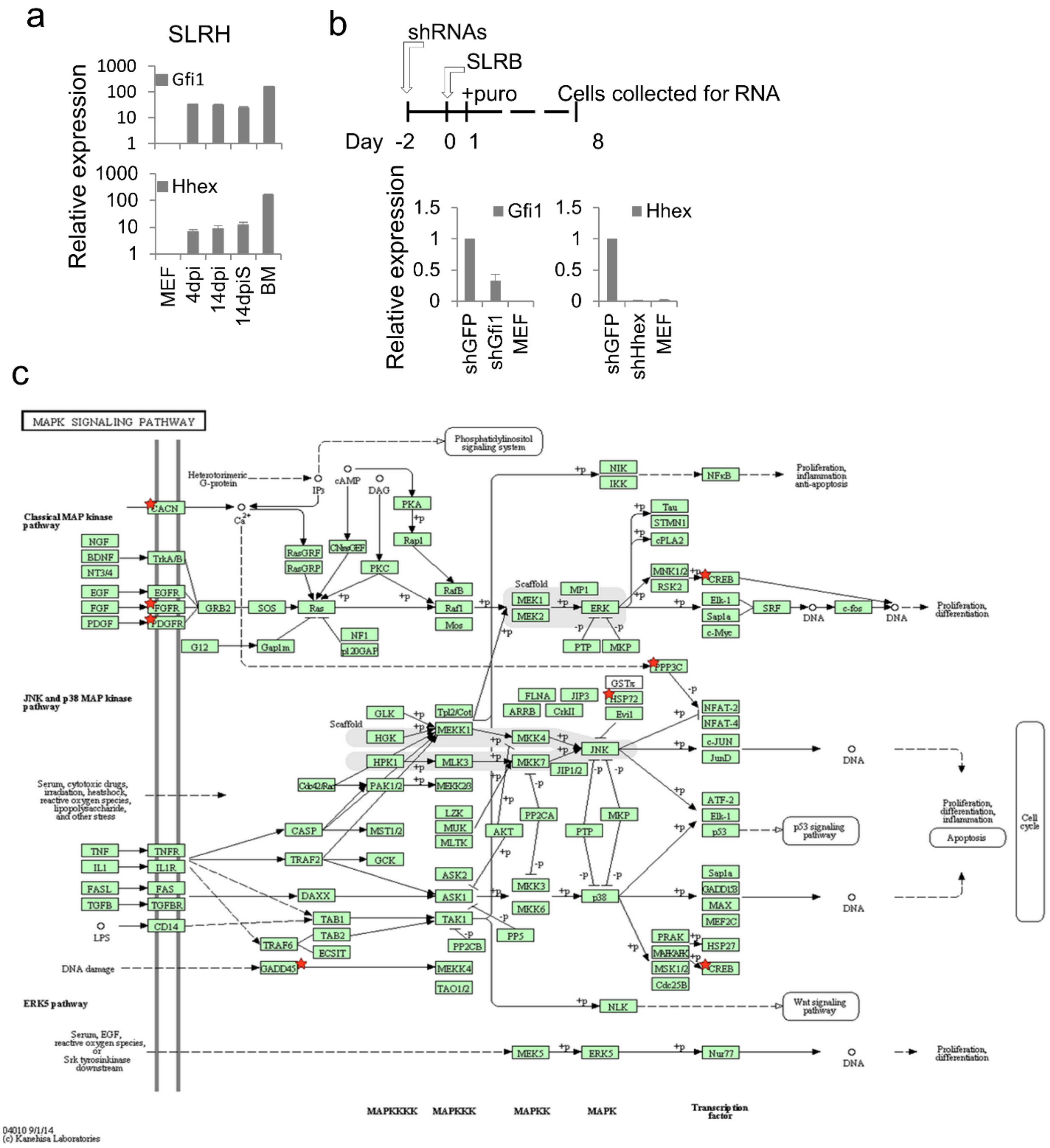

Supplementary Figure 9: 4dpi Lmo2-bound genes that are 4dpi/14dpi upregulated

(**a**) Validation of expression of Lmo2-bound genes *Gfi1* and *Hhex* at early stage of iHP reprogramming. tdTomato+ MEF were infected by SLRH and cells were collected for RNA analysis at indicated time course. 14dpi are from SLRH infected tdTomato+ cells at 14dpi without OP9 co-culture, 14dpiS are from sorted SLRH infected tdTomato+ cells from OP9 co-culture. Polycistronic SLR was used. Data shown as mean ± SD of technical triplicate. These are representative of two experiments.

(**b**) Evaluation of knockdown efficiency of shRNAs constructs of *Gfi1* and *Hhex*. A combination of ShGfi1-1& -2 (two shRNA constructs) or shHhex-1 & -2 (two shRNA constructs) were used for experiments, respectively. GFP shRNA was used as non-targeting control. Data shown are mean ± SD of technical triplicates. Polycistronic SLR was used. These are representative of two independent experiments.

(**c**) MAPK pathway map generated using DAVID. Genes, which are bound by Lmo2 and upregulated at 14 dpi cells, were uploaded to DAVID. The KEGG pathways enriched in these genes were identified. The red stars denote identified genes and their role in the pathway.

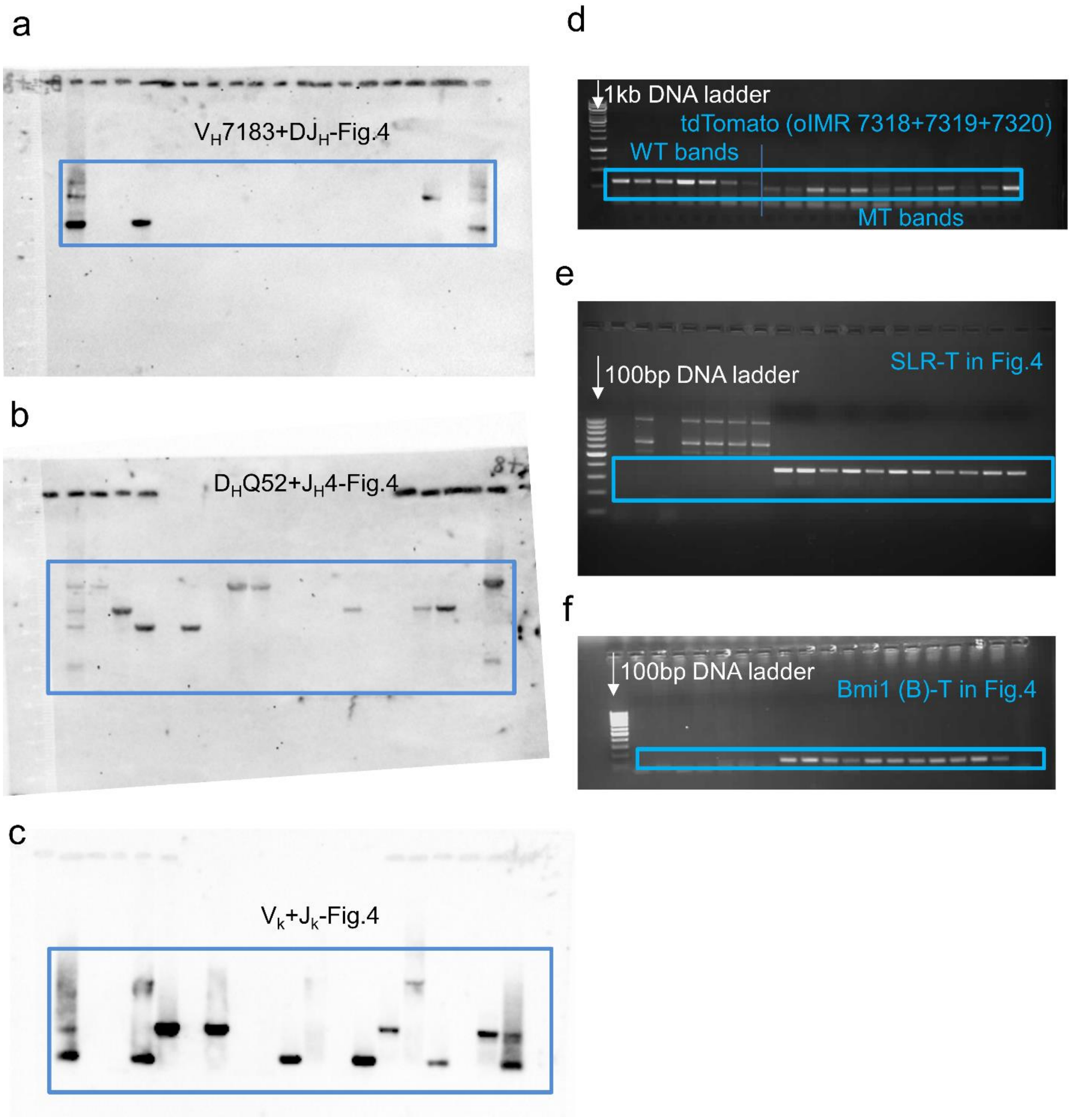

Supplementary Figure 10: Full blots and gel pictures used in Fig.4

a-**c**) Full southern blots used in Fig.4

c-**d**) Full gel pictures used in Fig.4

Supplementary Table 1. Representative genes that 4 dpi Lmo2 bound and up regulated at 4 dpi or 14 dpi during iHP reprogramming

Genes	**Distance to TSS	*D4/14 vs D0 (FC)	Genes	**Distance to TSS	*D14 vs D0 (FC)
Gfi1	-33501	1.95*	***Bmp4***	2557	5.72
Hhex	-379	1.20*	***Gab1***	672	2.35
Gata2	4534	1.79	***Wnt5a***	14352	2.72
Tcf3	-285	2.07	***Bmpr1a***	-5811	1.88
CD41	-263	1.36	***Tgfbr1***	-10486	1.40
Ebf3	-7223	1.12	***Tgfbr2***	-43490	1.80
Lgr5	1424	1.97	***Wisp1***	-29523	1.61
Egr1	26830	1.78	***Dkk3***	20445	1.49
Csf2ra	20710	1.49	***Vegfa***	69099	1.35
Fos	12950	6.09	***Angpt1***	-628	1.16
Nfib	-5203	3.13	***Stat1***	-5526	2.02
Fosb	-6751	2.81	***Stat3***	-210	1.14
Igf1	53	8.18	***Notch1***	-28213	1.46

* microarray data: D4 vs D0 are in blue, D14 vs D0 are in black (FC: fold change)
** 4dpi Lmo2 ChiP data: distance of 4dpi Lmo2 binding site from the transcriptional starting site (TSS) of the gene

Supplementary Table 2. Primers for cloning, genotyping, qPCR and V(D)J recombination

**Primers for cloning**	Sequence (5' -> 3')
Scl_Fw (BamHI/PacI))	GCCTGGAGAAGGATCCTTAATTAAGCCACCATGACGGAGCGGCCGCCGA
Scl_F2A_Rv	TCCCGCCAACTTGAGAAGGTCAAAATTCAAAGTCTGTTTCACGCCAGAACCCCGGGGGCCAGCCCCATCA
F2A_LMO2_Fw	TCTCAAGTTGGCGGGAGACGTGGAGTCCAACCCAGGGCCCATGTCCTCGGCCATCGAAA
LMO2_T2A_Rv (BamHI/ EcoRI)	CGGGATCCAGAATTCTTGGGCCAGGATTCTCCTCGACGTCACCGCATGTTAGCAGACTTCCTCTGCCCTCTCCGGAGCCTATCATCCCATTGATCTTAGTCCA
**shRNA target sequences**	
shGfi1-1	GCAAAGCTCATCATGGTTA
shGfi1-2	GGAACCTCGTTCCTTTGAA
shHhex-1	GCCTTAACTCCAAAGCCAT
shHhex-2	GCCCAGTGAACAGAATAAA
shGFP	GTTCACCTTGATGCCGTTC
**Primers for qPCR**	
β-major globin Fw	CACAACCCCAGAAACAGACA
β-major globin Rv	CTGACAGATGCTCTCTTGGG
ε-globin Fw	GGAGAGTCCATTAAGAACCTAGACA
ε-globin Rv	CTGTGAATTCATTGCCGAAGTGAC
βH1 Fw	CTCAAGGAGACCTTTGCTCA
βH1 Rv	AGTCCCCATGGAGTCAAAGA

Gfi1 Fw	AGAAGGCGCACAGCTATCAC
Gfi1 Rv	GGCTCCATTTTCGACTCGC
Hhex Fw	CGGACGGTGAACGACTACAC
Hhex Rv	CGTTGGAGAACCTCACTTGAC
Gapdh Fw	TGGCAAAGTGGAGATTGTTGCC
Gapdh Rv	AAGATGGTGATGGGCTTCCCG
Scl-Total (To)-Fw	CACTAGGCAGTGGGTTCTTTG
Scl-(To)-Rv	GGTGTGAGGACCATCAGAAATCT
Scl-(Endogenous, Endo)-Fw	CGGTGATGCGTCTGGGGCTG
Scl-(Endo)-Rv	ACCTGCCCTGAAGCCCAGCA
Lmo2-To)-Fw	GGAGAGACTATCTCAGGCTTTTTGG
Lmo2-(To)-Rv	ATCCGCTTGTCACAGGATGC
Lmo2-(Endo)-Fw	ATCTAGGCCAGAGTCCCAGGCAC
Lmo2-(Endo)-Rv	GTGAAGACCACACCGAGACGGC
Runx1-(To)-Fw	CGAAGACATCGGCAGAAACT
Runx1-(To)-Rv	GAGGCTGAGGGTTAAAGGCA
Runx1-(Endo)-Fw	GCGGCCCTACTGAGCTGAGC
Runx1-(Endo)-Rv	TCCAAGGGCCCTCCTGGCG
HoxB4-(To)-Fw	GTGAGCACGGTAAACCCCAAT
HoxB4-(To)-Rv	CGAGCGGATCTTGGTGTTG
HoxB4-(Endo)-Fw	AGTAGGAGGGCTTTCGGAAACAGGA
HoxB4-(Endo)-Rv	CGGAGGGAACTTGGGGTCGACATA
**Primers for transgenes (for both qPCR and integration)**	
pMx-1811s (paired with below	GACGGCATCGCAGCTTGGATACAC

gene Rv)	
LNCX 5' (For FUW vector paired with gene Rv)	AGCTCGTTTAGTGAACCGTCAGATC
hLmo2-721Rv:	GCTTCTGACAGGCGGCGCAT
mScl-2180Rv (for Scl-T and SLR-T)	AGCTTCGGCTGCTACTGCGTCG
hRunx1-2225Rv	AGCGATGGGCAGGGTCTTGTTGCAG
hBmi1-1943Rv	GATTCTCGTTGTTCGATG
**Primers for tdTomato genotyping**	
oIMR7318	CTCTGCTGCCT CTGGCTTCT
oIMR7319	CGAGGCGGATCACAAGCAATA
oIMR7320	TCAATG GGCGGGGGTCGTT
**Primers for PCR of V(D)J recombination**	
V_H7183	GTGGAGTCTGGGGGAGGCTTA
D_HQ52	CCACAGGCTCGAGAACTTTAGCG
J_H4e/R	AGGCTCTGAGATCCCTAGACAG
VκF	GGCTGCAGSTTCAGTGGCAGTGGRTCWGGRAC
Jκ5R	ATGCGACGTCAACTGATAATGAGCCCTCTCC
**Internal probes for PCR of V(D)J products**	
J_H4 probe	CTATGGACTACTGGGGTCAAGGAAC
Upstream Jκ5 probe	ACCAAGCTGGAGCTGAAACGTAAGTACAC
